# Supplementary material for: Guidance for reporting intervention development studies in health research (GUIDED): an evidence-based consensus study
Source: BMJ Open. 2020 Apr 8;10(4):e033516. doi: 10.1136/bmjopen-2019-033516 (PMC7245409; doi:10.1136/bmjopen-2019-033516)
Supplement: Author's manuscript [file bmjopen-2019-033516.draft_revisions.pdf]

BMJ Open

BMJ Open is committed to open peer review. As part of this commitment we make the peer review history of every article we publish publicly available.

When an article is published we post the peer reviewers' comments and the authors' responses online. We also post the versions of the paper that were used during peer review. These are the versions that the peer review comments apply to.

The versions of the paper that follow are the versions that were submitted during the peer review process. They are not the versions of record or the final published versions. They should not be cited or distributed as the published version of this manuscript.

BMJ Open is an open access journal and the full, final, typeset and author-corrected version of record of the manuscript is available on our site with no access controls, subscription charges or pay-per-view fees (http://bmjopen.bmj.com).

If you have any questions on BMJ Open's open peer review process please email info.bmjopen@bmj.com

BMJ Open

Reporting GUIDance for intervEntion Development in health research (GUIDED)

Journal:	*BMJ Open*
Manuscript ID	bmjopen-2019-033516
Article Type:	Research
Date Submitted by the Author:	08-Aug-2019
Complete List of Authors:	Duncan, Edward; University of Stirling, NMAHP-RU<br>O'Cathain, Alicia; University of Sheffield, School of Health and Related Research<br>Rousseau, Nikki; University of Stirling<br>Sworn, Katie; University of Sheffield, Health Economics and Decision Science, School of Health and Related Research<br>Turner, Katrina; University of Bristol, School of Social and Community Medicine<br>Yardley, Lucy; University of Southampton, Psychology; University of Bristol, School of Health Sciences<br>Hoddinott, Pat; University of Stirling, Nursing, Midwifery and Allied Health Professional Research Unit
Keywords:	Intervention development, Reporting Guidance, Delphi, Consensus

SCHOLARONE™
Manuscripts

BMJ

I, the Submitting Author has the right to grant and does grant on behalf of all authors of the Work (as defined in the below author licence), an exclusive licence and/or a non-exclusive licence for contributions from authors who are: i) UK Crown employees; ii) where BMJ has agreed a CC-BY licence shall apply, and/or iii) in accordance with the terms applicable for US Federal Government officers or employees acting as part of their official duties; on a worldwide, perpetual, irrevocable, royalty-free basis to BMJ Publishing Group Ltd ("BMJ") its licensees and where the relevant Journal is co-owned by BMJ to the co-owners of the Journal, to publish the Work in this journal and any other BMJ products and to exploit all rights, as set out in our licence.

The Submitting Author accepts and understands that any supply made under these terms is made by BMJ to the Submitting Author unless you are acting as an employee on behalf of your employer or a postgraduate student of an affiliated institution which is paying any applicable article publishing charge ("APC") for Open Access articles. Where the Submitting Author wishes to make the Work available on an Open Access basis (and intends to pay the relevant APC), the terms of reuse of such Open Access shall be governed by a Creative Commons licence – details of these licences and which Creative Commons licence will apply to this Work are set out in our licence referred to above.

Other than as permitted in any relevant BMJ Author's Self Archiving Policies, I confirm this Work has not been accepted for publication elsewhere, is not being considered for publication elsewhere and does not duplicate material already published. I confirm all authors consent to publication of this Work and authorise the granting of this licence.

For peer review only

Reporting GUIDance for intervEntion Development in health research (GUIDED)

*Edward AS Duncan, NMAHP Research Unit, University of Stirling, FK9 4LA, Stirling, Scotland, UK Associate Professor

edward.duncan@stir.ac.uk Tel. 01786 46 6286

Alicia O'Cathain School of Health and Related Research (ScHARR), University of Sheffield, Professor

a.ocathain@sheffield.ac.uk

Nikki Rousseau NMAHP Research Unit, University of Stirling, Research Fellow

nikki.rousseau@newcastle.ac.uk

Liz Croot, School of Health and Related Research (ScHARR), University of Sheffield, Senior Research Fellow

l.croot@sheffield.ac.uk

Katie Sworn, School of Health and Related Research (ScHARR), University of Sheffield, Research Associate

k.sworn@sheffield.ac.uk

Katrina Turner, Population Health Sciences, University of Bristol, Reader

Katrina.Turner@bristol.ac.uk

Lucy Yardley, School of Psychological Sciences, University of Bristol, Professor

lucy.yardley@bristol.ac.uk

Pat Hoddinott, NMAHP Research Unit, University of Stirling, Professor

p.m.hoddinott@stir.ac.uk

*Corresponding author

The Corresponding Author has the right to grant on behalf of all authors and does grant on behalf of all authors, an exclusive licence on a worldwide basis to the BMJ Publishing Group Ltd to permit this article (if accepted) to be published in BMJ editions and any other BMJPGL products and sub-licences such use and exploit all subsidiary rights, as set out in our licence.

Word Count = 3,551

For peer review only - http://bmjopen.bmj.com/site/about/guidelines.xhtml

Abstract

Objective: To improve the quality and consistency of intervention development reporting in health research.

Design: A consensus exercise consisting of two simultaneous and identical three round e-Delphi studies (one with experts in intervention development and one with wider stakeholders including funders, journal editors and public involvement members), followed by a consensus workshop. E-Delphi items were systematically derived from two preceding systematic reviews and a qualitative interview study.

Participants: Intervention developers (n=26) and wider stakeholders (n=18) from the UK, North America and Europe participated in separate e-Delphi studies. Intervention developers (n=13) and wider stakeholders (n=13) participated in a one day consensus workshop.

Results: e-Delphi participants achieved consensus on 14 reporting items. Following feedback from the consensus meeting, the final inclusion and wording of 13 items with description and explanations for each item were agreed. Items focus on context, purpose, target population, approaches, evidence, theory, guiding principles, stakeholder contribution, changes in content or format during the development process, required changes for sub-groups, continuing uncertainties, and open-access publication. They form the GUIDED checklist.

Conclusions: Consensus-based reporting guidance for intervention development in health research is now available for publishers and researchers to use. GUIDED has the potential to lead to greater transparency, and enhance quality and improve learning about intervention development research and practice.

Article Summary

Strengths and Limitations of this Study:

- The 14 GUIDED items were developed through a structured and transparent consensus-based process.
- Parity of opinion was given to intervention developers and wider stakeholders throughout the development of the reporting guidance.
- Despite aiming to secure an international sample this proved difficult. We acknowledge that participants in the study were based in the Global North and that the perspectives of intervention developers and wider stakeholders form the Global South is absent.
- As intervention development becomes further understood over time, it is likely that the reporting guidance will need to be reconsidered.

Key words: Intervention Development, Reporting Guidance, Delphi, Consensus

For peer review only - http://bmjopen.bmj.com/site/about/guidelines.xhtml

Background

The United Kingdom Medical Research Council's (MRC) Framework for the Development and Evaluation of Complex Interventions(1) lists intervention development as the first of a series of interconnected phases. While the MRC complex intervention guidance has stimulated considerable methodological progress in understanding and reporting the latter phases (i.e. feasibility and piloting, evaluation, and implementation), the intervention development phase has remained relatively underdeveloped and without a comprehensive reporting guideline(2). Research funders, researchers, commissioners, practitioners, the public and patients are increasingly interested in understanding and improving the intervention development process.

There are a variety of ways to develop interventions. A recent review identified various approaches including, partnership (e.g. co-production; co-design), target population centred, evidence and theory-based, implementation-based, efficiency-based, step or phased-based, intervention-specific or a combination of methods(3). Successful intervention development is characterised as being rigorous, scientific and resulting in effective interventions that can be implemented in real world settings(4). However, a key intervention development challenge is the lack of evidence-based quality criteria on which to assess which, if any, approach is superior to another, and in which context.

Intervention development processes are currently under-reported. This may be due to research funding priorities or pressure to publish efficacy or effectiveness studies diminishing the priority of publishing intervention development studies. When intervention development studies are published, they are sometimes included as part of a feasibility or pilot study. Consequently, detail about how the intervention was actually developed can be sparse. A more systematic, comprehensive, and transparent approach to intervention development reporting is likely to enhance understanding about the intervention development process. It would help readers to understand the benefits and challenges of different intervention development approaches. It would help researchers select an intervention development approach that is relevant to their context. It would also facilitate future retrospective assessment of how different intervention development approaches can lead to either effective or ineffective interventions that do or do not translate into practice change. Potentially such assessment could provide insights into research waste. While the TIDiER(5) guidelines already exist these relate to intervention description, not intervention development. The CREDICI 2 (6) guideline for reporting the development and evaluation of Complex Interventions in Healthcare does provide reporting guidance for intervention development, however this is limited to four items, as CREDECI 2 also provides guidance on reporting Feasibility and Piloting and Evaluation. To date, there has been no guidance focusing in detail on reporting the whole process of intervention development.

This paper presents GUiDance for the rEporting of Intervention Development (GUIDED). GUIDED forms part of a larger MRC funded study to produce guidance on intervention development: the IdentifyiNg and assessing different approaches to DEveloping compleX interventions (INDEX) study(7). GUIDED is the first international mixed methods consensus study to focus on reporting guidance soley for intervention development processes. In this paper we report the methods used to develop and gain consensus on the items included in the GUIDED checklist. We present each reporting item with further description and explanation. GUIDED will be of interest to research funders, researchers, journal editors, commissioners, practitioners, the public and patients, who we refer to collectively as "readers".

For peer review only - http://bmjopen.bmj.com/site/about/guidelines.xhtml

Methods

Design

We published our intent to develop intervention reporting guidance (5 July 2017) in the Equator Network Library (http://www.equator-network.org/library/reporting-guidelines-under-development/reporting-guidelines-under-development-for-other-study-designs/#80). The design of this intervention development consensus study involved conducting two simultaneous and identical e-Delphi studies followed by a consensus workshop. Participants included i) intervention developers and ii) wider stakeholders who were involved in the wider intervention development activities including directors of research funding panels, editors of journals that had published intervention development studies, public and patient involvement members of intervention development studies, and people working in health service implementation(7). By separating intervention developers and wider stakeholders within the e-Delphi process we ensured that the perceptions of both groups were equally reported and their views given equal weight. A subset of the consensus exercise related specifically to the identification of intervention development reporting guidance, reported in this paper. We followed established methods for developing reporting guidance(8) (See Figure 1) and report the e-Delphi guidance in line with current best practice(9). The parallel e-Delphi studies were delivered over three separate rounds. Each round lasted for 4 weeks. Non-responders were emailed a reminder after two weeks. Completion of one round was required to enter the next e-Delphi round. There was space for participants to comment beside each item and explain their responses, or (in Round 1) suggest alternative item wording. However no additional items were suggested by participants.

e-Delphi item generation

e-Delphi items were generated by triangulating three different data sources: a systematic methods overview of 87 articles, books and websites that identified 23 approaches to intervention development within 8 categories and with 18 actions undertaken across these approaches(3); a systematic review of 87 international primary research articles reporting intervention development processes which describes 10 actions(10); and an analysis of 21 in-depth qualitative interviews with an international sample of intervention developers (N=15) and key stakeholders (N=6) (11). The research team (AO, LY, PH, ED, LC, NR, KS) met regularly to identify the potential reporting guidance items. Members of the research team worked in pairs, and one team of three, to extract potential guidance items from the three data sources. Each pair then presented potential e-Delphi items to the whole team. Each potential item was discussed, refined, and agreed. We grouped items into themes, with one theme entitled "Reporting Guidance", which had 14 items. The full set of e-Delphi items and their ratings have been reported elsewhere(7).

e-Delphi participants

Invitations were sent to 92 individuals who had undertaken intervention development and/or published a formal approach to intervention development and 80 wider stakeholders. Individuals who responded to say they would participate in the e-Delphi were emailed a study information containing a url to an established e-Delphi platform (12) and a unique password to access the study. Informed consent was provided by participants as part of the e-Delphi platform registration process.

For peer review only - http://bmjopen.bmj.com/site/about/guidelines.xhtml

Definition of consensus

Following an online consent process, participants were asked to rate the importance they would give to each potential item on a scale of 1 to 5: Not at all important (1); Slightly unimportant (2); Somewhat important (3); Fairly important (4); Very important (5). An additional option of no relevant expertise was provided for each item. Prior to commencement, we decided that an item would be included within the reporting guidance if at least 70% of participants agreed that an item was fairly important (4) or very important (5) in either e-Delphi group by the end of Round 3.

Consensus meeting

The results of the e-Delphi studies were discussed by participants (in person or by video link) and eight team members at a one day consensus meeting on 13 March 2018, in London, UK. The meeting began with presentations from the team: an overview of the overall INDEX study, followed by a summary overview of both systematic reviews(3, 10) and qualitative study(11). The results of both e-Delphi studies were then presented and detailed discussions were held on items that had not reached consensus, but which reflected divergence. Consensus meeting participants suggested improvements to the wording of items. Two items were merged in the final GUIDED checklist: Item 10 "Report how the intervention changed in content and format from the start of the intervention development process" and a recommendation to "report the reasons for discarding intervention components that were considered". This was previously a separate item but was considered by the team to be appropriately covered in a single item.

*** Insert Figure 1 about here ***

Patient and Public Involvement

Patient and Public Involvement (PPI) members were invited to an expert panel meeting held at the start of the INDEX study, and to a consensus conference following the e-Delphi studies. None attended the first meeting, but PPI members did attend the consensus conference. All study participants will be sent copies of journal publications relating to the INDEX study.

Results

Description of participants

The response rates for each round were as follows: Round 1 Intervention developers (n=34), Wider stakeholders (n=22); Round 2 Intervention developers (n=27), Wider stakeholders (n=18); Round 3 Intervention developers (n=26), Wider stakeholders (n=18). Intervention development participants who completed Round 3 were based in the UK (n=16), mainland Europe (n=5), Ireland (n=4) and USA (n=1). They included people from public health (n=10), applied health research/health services research (n=8), psychology (n=7), nursing (n=6), and allied health professional (n=1) backgrounds. Wider stakeholder participants who completed Round 3 were based in the UK (n=16), mainland Europe (n=1) and USA (n=1). They included chairs or members of funding panels (n=5), editors or editorial board members of journals (n=4), commissioners of services (n=3), public and patient involvement (n=3), and other (n=3) individuals. All fourteen intervention development reporting items reached our a priori threshold for inclusion (Table 1)(7).

For peer review only - http://bmjopen.bmj.com/site/about/guidelines.xhtml

The 26 participants of the consensus meeting were based in the UK (n=19), USA (n=3), mainland Europe (n=3), and Ireland (n=1). They were invited due to their varied roles in the intervention development process: intervention developers (n=13), methodologists (n=4), chairs of funding panel (n=3), journal editors (n=3), public and patient representatives (n=1), commissioner (n=1), and other (n=1).

Description of consensus from survey

Table 1 presents all the reporting items included in the e-Delphi; the percentage of responses that scored 4 or 5 for each item and the mode score.

Table 1. e-Delphi study results for Intervention Development reporting items.

**Item** (presented in order of degree of consensus)	**Intervention Developers**		**Wider Stakeholders**	
	Mode Score	% Agree	Mode Score	% Agree
Report the purpose of the intervention	5	100	5	100
Report the target population	5	100	5	100
Report any use of components from an existing intervention	5	100	4	100
Report how evidence from different sources informed the intervention development	5	100	5	100
Report how stakeholders contributed to the intervention development process	5	100	4	94
Report important uncertainties at the end of the intervention development process.	5	100	5	78
Report the context for which the intervention was developed	5	96	5	100
Report any changes to interventions required or likely to be required for subgroups	5	96	4	83
Report how any published intervention development approach contributed to the development process	5	92	4	71
Report how existing published theory informed the intervention development process	5	92	5	94
Report any guiding principles, people or factors which were prioritised when making decisions	5	92	4	83
Report how the intervention changed in content and format from the start of the intervention development process*	5	88	4	94
Report the reasons for discarding intervention components that were considered*	5	88	4	88
Report the intervention development in an open access format (e.g. open access journal, report chapter, website)	4	77	5	89

*These items were merged into one item (See Item 10 below) following the consensus meeting.

GUIDED intervention development reporting items: Description and explanation

Below, we have ordered the items so that those which are more likely to be considered earlier in the development process are listed first. However, there is no fixed order in which the reporting items must be considered. EQUATOR reporting guidance(8) encourages describing and explaining the rationale for each reporting item to help researchers and others to write or appraise reports. We have therefore followed this format, in keeping with other related reporting guidelines(5, 13, 14).

For peer review only - http://bmjopen.bmj.com/site/about/guidelines.xhtml

A blank checklist to support the use of GUIDED by authors and reviewers is provided in Supplementary File 1. Examples from papers that meet a reporting item specification are provided in Supplementary file 2. A full copy of the guidance with checklist is available from the EQUATOR Network (www.equator-network.org).

Item 1.

Description: Report the context for which the intervention was developed.

Explanation: Understanding the context in which an intervention was developed informs readers about the suitability and transferability of the intervention to the context in which they are considering evaluating, adapting or using the intervention. Context here can include place, organisational and wider socio-political factors that may influence the development and/or delivery of the intervention (15).

Item 2.

*Description***:** Report the purpose of the intervention development process.

Explanation: Clearly describing the purpose of the intervention specifies what it sets out to achieve. The purpose may be informed by research priorities, for example those identified in systematic reviews, evidence gaps set out in practice guidance such as The National Institute for Health and Care Excellence or specific prioritisation exercises such as those undertaken with patients and practitioners through the James Lind Alliance.

Item 3.

Description: Report the target population for the intervention development process.

Explanation: The target population is the population that will potentially benefit from the intervention – this may include patients, clinicians, and/or members of the public. If the target population is clearly described then readers will be able to understand the relevance of the intervention to their own research or practice. Health inequalities, gender and ethnicity are features of the target population that may be relevant to intervention development processes.

Item 4.

Description: Report how any published intervention development approach contributed to the development process.

Explanation: Many formal intervention development approaches exist and are used to guide the intervention development process (e.g. 6Squid(16) or The Person Based Approach to Intervention Development(17)). Where a formal intervention development approach is used, it is helpful to describe the process that was followed, including any deviations. More general approaches to intervention development also exist(3) and have been categorised as follows:- Target Population-centred intervention development; evidence and theory-based intervention development; partnership intervention development; implementation-based intervention development; efficacy-based intervention development; step or phased-based intervention development; and intervention-specific intervention development. These approaches do not always have specific guidance that describe their use. Nevertheless, it is helpful to give a rich description of how any published approach was operationalised.

Item 5.

For peer review only - http://bmjopen.bmj.com/site/about/guidelines.xhtml

Description: Report how evidence from different sources informed the intervention development process.

Explanation: Intervention development is often based on published evidence and/or primary data that has been collected to inform the intervention development process. It is useful to describe and reference all forms of evidence and data that have informed the development of the intervention because evidence bases can change rapidly, and to explain the manner in which the evidence and/or data was used. Understanding what evidence was and was not available at the time of intervention development can help readers to assess transferability to their current situation.

Item 6.

Description: Report how/if existing published theory informed the intervention development process

Explanation: Reporting whether and how theory informed the intervention development process aids the reader's understanding of the theoretical rationale that underpins the intervention. Though not mentioned in the e-Delphi or consensus meeting, it became increasingly apparent through the development of our guidance that this item could relate to either existing published theory or programme theory.

Item 7.

Description: Report any use of components from an existing intervention in the current intervention development process.

Explanation: Some interventions are developed with components that have been adopted from existing interventions. Clearly identifying components that have been adopted or adapted and acknowledging their original source helps the reader to understand and distinguish between the novel and adopted components of the new intervention.

Item 8.

Description: Report any guiding principles, people or factors that were prioritised when making decisions during the intervention development process.

Explanation: Reporting any guiding principles that governed the development of the intervention will help the reader to understand the authors' reasoning behind the decisions that were made. Guiding principles specify the core objectives and features of the desired intervention(18). These could include prioritising patient preferences over clinician preferences, providing an engaging experience for patients, minimizing the cost of delivering the intervention, or maximising the potential for the intervention to be scaled up.

Item 9.

Description: Report how stakeholders contributed to the intervention development process.

Explanation: Potential stakeholders can include patient and community representatives, local and national policy makers, health care providers and those paying for or commissioning health care. Each of these groups may influence the intervention development process in different ways. Specifying how differing groups of stakeholders contributed to the intervention development process helps the reader to understand how stakeholders were involved and the degree of influence they had on the overall process. Further detail on how to integrate stakeholder contributions within intervention reporting are available (19).

For peer review only - http://bmjopen.bmj.com/site/about/guidelines.xhtml

For peer review only

Item 10.

Description: Report how the intervention changed in content and format from the start of the intervention development process.

Explanation: Intervention development is frequently an iterative process. The conclusion of the initial phase of intervention development does not necessarily mean that all uncertainties have been addressed. It is helpful to list remaining uncertainties such as the intervention intensity, mode of delivery, materials, procedures, or type of location that the intervention is most suitable for. This can guide other researchers to potential future areas of research and practitioners about uncertainties relevant to their healthcare context.

Item 11.

Description: Report any changes to interventions required or likely to be required for subgroups.

Explanation: Specifying any changes that the intervention development team perceive are required for the intervention to be delivered or tailored to specific sub groups enables readers to understand the applicability of the intervention to their target population or context. These changes could include changes to personnel delivering the intervention, to the content of the intervention, or to the mode of delivery of the intervention.

Item 12.

Description: Report important uncertainties at the end of the intervention development process.

Explanation: Intervention development is frequently an iterative process. The conclusion of the initial phase of intervention development does not necessarily mean that all uncertainties have been addressed. It is helpful to list remaining uncertainties such as the intervention intensity, mode of delivery, materials, procedures, or type of location that the intervention is most suitable for. This can guide other researchers to potential future areas of research and practitioners about uncertainties relevant to their healthcare context.

Item 13.

Description: Report the intervention development process in an open access format.

Explanation: Unless reports of intervention development are available people considering using an intervention cannot understand the process that was undertaken and make a judgement about its appropriateness to their context. It also limits cumulative learning about intervention development methodology and observed consequences at later evaluation, translation and implementation stages. Reporting intervention development in an open access (Gold or Green) publishing format increases the accessibility and visibility of intervention development research and makes it more likely to be read and used. Potential platforms for open access publication of intervention development include open access journal publications, freely accessible funder reports, or a study web-page that details the intervention development process.

Discussion

Intervention development is a vital, but under-reported, component of the MRC Developing and evaluating complex interventions guidance. This study presents a mixed method international consensus study to produce detailed reporting guidance for the intervention development phase of the MRC Complex Intervention Guidance. The GUIDED checklist provides a list of 13 intervention development items each with an accompanying explanation for why it is important to include this

For peer review only - http://bmjopen.bmj.com/site/about/guidelines.xhtml

information in publications and outputs that describe the intervention development process. The GUIDED checklist was developed in collaboration with a range of stakeholders, each of whom contributed a range of expertise and perspectives on the intervention development process.

The GUIDED reporting checklist and its associated item descriptions have been systematically developed to support readers to understand key aspects of specific intervention development studies. Adhering to the GUIDED item checklist across the variety of formats in which intervention development publications already occur should improve the quality, transparency and consistency of intervention development reporting.

To our knowledge, this is the first international mixed-method consensus study purely relating to reporting intervention development studies. We struggled to recruit individuals working outside of the UK, and in particular from individuals who work in the Global South. This could limit the transferability of our findings and reporting guidance. The thinking of the research team developed over the course of the study. One example of this relates to the description of theory. The agreed e-Delphi item related solely to existing published theory. However, towards the end of the study is became apparent to the research team that this item should have explicitly included programme theory within the item description. We therefore accounted for this within the item explanation. As intervention development becomes further understood over time, it is likely that the reporting guidance will need to be reconsidered.

What gap does GUIDED fill?

Good quality effectiveness studies with detailed guidance on intervention description are necessary(5, 13). GUIDED is offered as complementary reporting guidance to detail the intervention development process. Presenting intervention development studies in line with the GUIDED items reported in this paper will enable commissioners and practitioners to understand the context and methods that were used to develop the intervention to help them make judgements about the quality and relevance of the intervention. This information will be useful in guiding their decisions about whether to evaluate or implement an intervention within their specific context. Finally, high quality and transparent reporting of intervention development in line with GUIDED recommendations will enable methodological lessons to be learnt and incorporated into future intervention development studies. We therefore recommend that authors follow GUIDED when reporting intervention development studies, and journal editors and research funders endorse the use of GUIDED within any publications that report intervention development studies. The GUIDED checklist will be placed on the EQUATOR network website and we request that Journals provide links to the EQUATOR site and signpost potential authors to this guidance where appropriate.

How does GUIDED fit with other reporting guidance?

GUIDED provides a more comprehensive description than previous guidance(6) of what should be reported when publishing intervention development studies. GUIDED complements and can easily be integrated or signposted to within other reporting guidance. Papers that are written to describe interventions should follow existing guidance(5, 13) and signpost readers to where they can read about the intervention development process, reported in line with GUIDED recommendations, so they can judge the appropriateness of the intervention development process. Where randomised controlled trials are being reported using CONSORT guidance(14) then authors could signpost (for example in reporting CONSORT Statement 5: Intervention) to where a GUIDED description of intervention development has been reported. Where patients and the public contributed to intervention development, the GRIPP 2 guidelines can be used(19).

For peer review only - http://bmjopen.bmj.com/site/about/guidelines.xhtml

Conclusion

The GUIDED checklist and reporting guidance has been developed by following internationally recognised methods for developing reporting guidance(8), with items based on extensive primary(11, 20) and secondary(3, 10) research to enable greater transparency and quality of reporting development of complex interventions. The GUIDED checklist and guidance provide a clear and structured basis for the reporting of intervention development studies in a range of formats. It has the potential to facilitate learning about how early intervention development decisions impact across the life history of an intervention: through feasibility and efficacy testing, cost-effectiveness evaluations and translation into health care practice change.

Funding: this work was supported by MRC-NIHR Methodology Research Panel, grant number MR/N015339/1. Funders had no influence on the reporting guidance presented here. The authors were fully independent of the funders.

Competing interests: All authors have read and understood BMJ Group policy on declaration of interests and have no relevant interests to declare.

Ethics committee approval: Ethical consent to undertake the study was secured from the University of Stirling, General University Ethics Panel (GUEP37).

Authors contributions: ED led the e-Delphi studies, working with NR, and co-led the consensus event with LY. ED wrote the first draft of the paper. All authors substantially contributed to developing the initial e-Delphi items and the consensus event. All authors discussed the issues addressed in the reporting guidance and commented on multiple drafts of the paper, including the final draft. ED acts as guarantor.

All authors have completed the Unified Competing Interest form (available on request from the corresponding author) and declare: financial support from Medical Research Council and NIHR for this study; no financial relationships with any organisations that might have an interest in the submitted work in the previous three years, no other relationships or activities that could appear to have influenced the submitted work.

Data sharing statement: All data relating to the reporting guidance is available upon request.

Acknowledgements: We wish to thanks the participants of both e-Delphis and the consensus conference. We wish to thank Kevin Swingler (Senior Lecturer, Computing Science and Mathematics, University of Stirling) for his management of the Stirling e-Delphi Platform during our e-Delphi studies).

For peer review only - http://bmjopen.bmj.com/site/about/guidelines.xhtml

References

1. Craig P, Dieppe P, Macintyre S, Michie S, Nazareth I, Petticrew M. Developing and evaluating complex interventions: the new Medical Research Council guidance. Bmj. 2008;337:a1655.
2. Hoddinott P. A new era for intervention development studies. BioMed Central; 2015.
3. O'Cathain A, Croot L, Sworn K, Duncan E, Rousseau N, Turner K, et al. Taxonomy of approaches to developing interventions to improve health: a systematic methods overview. 2019;5(1):41.
4. Turner KM, Rousseau N, Croot L, Duncan E, Yardley L, O'Cathain A, et al. Understanding successful development of complex health and healthcare interventions and its drivers from the perspective of developers and wider stakeholders: an international qualitative interview study. 2019;9(5):e028756.
5. Hoffmann TC, Glasziou PP, Boutron I, Milne R, Perera R, Moher D, et al. Better reporting of interventions: template for intervention description and replication (TIDieR) checklist and guide. Bmj. 2014;348:g1687.
6. Möhler R, Köpke S, Meyer GJT. Criteria for Reporting the Development and Evaluation of Complex Interventions in healthcare: revised guideline (CReDECI 2). 2015;16(1):204.
7. O'Cathain AC, E; Duncan, E; Rousseau, N; Sworn, K; Turner, K; Yardley, L; Hoddinott, P. Guidance on how to develop complex interventions to improve health and health care. BMJ Open. Submitted.
8. Moher D, Schulz KF, Simera I, Altman DG. Guidance for developers of health research reporting guidelines. PLoS medicine. 2010;7(2):e1000217.
9. Hasson F, Keeney S, McKenna HJJoan. Research guidelines for the Delphi survey technique. 2000;32(4):1008-15.
10. Croot L OCA, Sworn K, Duncan EAS, Rosseau N, Turner K, Yarldey L, Hoddinott P. How interventions to improve health are developed: a systematic review of international practice. BMC Pilot and Feasibility Studies. Submitted.
11. Turner K RN, O'Cathain A, Croot L, Duncan EAS, Sworn K, Yardley L, Hoddinott P. Successful intervention development and its drivers: a qualitative interview study. Submitted.
12. Duncan E, K S. The Stirling eDelphi Platform Stirling, UK: University of Stirling; 2015 [Available from: http://delphi.stir.ac.uk/overview.pdf.
13. Armstrong R, Campbell M, Craig P, Hoffmann T, Katikireddi SV, Waters E. Reporting guidelines for population health and policy interventions: TIDieR-PHP. The Lancet. 2015;386:S19.
14. Eldridge SM, Chan CL, Campbell MJ, Bond CM, Hopewell S, Thabane L, et al. CONSORT 2010 statement: extension to randomised pilot and feasibility trials. Pilot and feasibility studies. 2016;2(1):64.
15. Craig P, Di Ruggiero E, Frolich KL, Mykhalovskiy E, White M, Campbell R, et al. Taking account of context in population health intervention research: guidance for producers, users and funders of research. 2018.
16. Wight D, Wimbush E, Jepson R, Doi LJJECH. Six steps in quality intervention development (6SQuID). 2016;70(5):520-5.
17. Yardley L, Morrison L, Bradbury K, Muller IJJomIr. The person-based approach to intervention development: application to digital health-related behavior change interventions. 2015;17(1):e30.
18. Morrison L, Muller I, Yardley L, Bradbury KJTEHP. The person-based approach to planning, optimising, evaluating and implementing behavioural health interventions. 2018;20(3):464-9.
19. Staniszewska S, Brett J, Simera I, Seers K, Mockford C, Goodlad S, et al. GRIPP2 reporting checklists: tools to improve reporting of patient and public involvement in research. 2017;3(1):13.
20. Rousseau N, Turner, KM, Duncan EAS, O'Cathain A, Croot L, Yardley L, Hoddinott P. Attending to design when developing complex health interventions: a qualitative interview study with intervention developers and stakeholders. PLOS One. Submitted.

For peer review only - http://bmjopen.bmj.com/site/about/guidelines.xhtml

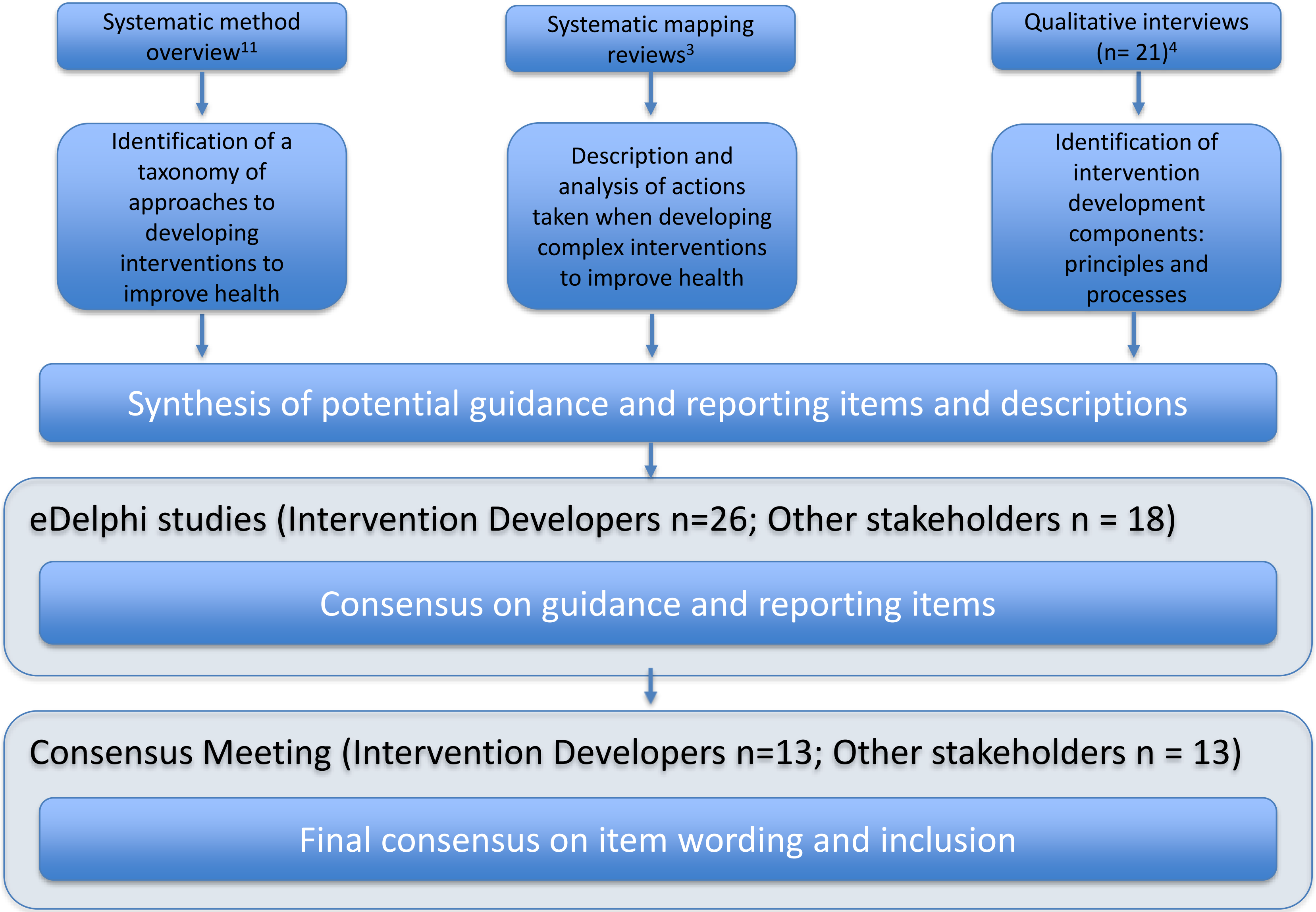
Systematic method overview[11]
Systematic mapping reviews[3]
Qualitative interviews (n= 21)[4]
Identification of a taxonomy of approaches to developing interventions to improve health
Description and analysis of actions taken when developing complex interventions to improve health
Identification of intervention development components: principles and processes
Synthesis of potential guidance and reporting items and descriptions
eDelphi studies (Intervention Developers n=26; Other stakeholders n = 18)
Consensus on guidance and reporting items
Consensus Meeting (Intervention Developers n=13; Other stakeholders n = 13)
Final consensus on item wording and inclusion

GUIDED – a guideline for reporting for intervention development studies.

Supplementary File 1: Blank Checklist

Item description	Explanation	Page in manuscript where item is located	Other*
1. Report the context for which the intervention was developed.	Understanding the context in which an intervention was developed informs readers about the suitability and transferability of the intervention to the context in which they are considering evaluating, adapting or using the intervention. Context here can include place, organisational and wider socio-political factors that may influence the development and/or delivery of the intervention (15).		
2. Report the purpose of the intervention development process.	Clearly describing the purpose of the intervention specifies what it sets out to achieve. The purpose may be informed by research priorities, for example those identified in systematic reviews, evidence gaps set out in practice guidance such as The National Institute for Health and Care Excellence or specific prioritisation exercises such as those undertaken with patients and practitioners through the James Lind Alliance.		
3. Report the target population for the intervention development process.	The target population is the population that will potentially benefit from the intervention – this may include patients, clinicians, and/or members of the public. If the target population is clearly described then readers will be able to understand the relevance of the intervention to their own research or practice. Health inequalities, gender and ethnicity are features of the target population that may be relevant to intervention development processes.		
4. Report how any published intervention development approach contributed to the development process	Many formal intervention development approaches exist and are used to guide the intervention development process (e.g. 6Squid (16) or The Person Based Approach to Intervention Development (17)). Where a formal intervention development approach is used, it is helpful to describe the process that was followed, including any deviations. More general approaches to intervention development also exist and have been categorised as follows (3):- Target Population-centred intervention development; evidence and theory-based intervention development; partnership intervention development; implementation-based intervention development; efficacy-based intervention development; step or phased-based intervention development; and intervention-specific intervention development (3). These approaches do not always have specific guidance that describe their use. Nevertheless, it is helpful to give a rich description of how any published approach was operationalised		
5. Report how evidence from different sources informed the intervention development process.	Intervention development is often based on published evidence and/or primary data that has been collected to inform the intervention development process. It is useful to describe and reference all forms of evidence and data that have informed the development of the intervention because evidence bases can change rapidly, and to explain the manner in which the evidence and/or data was used. Understanding what evidence was and was not available at the time of intervention development can help readers to assess transferability to their current situation.		
6. Report how/if published theory informed the intervention development process.	Reporting whether and how theory informed the intervention development process aids the reader's understanding of the theoretical rationale that underpins the intervention. Though not mentioned in the e-Delphi or consensus meeting, it became increasingly apparent through the development of our guidance that this theory item could relate to either existing published theory or programme theory		

For peer review only - http://bmjopen.bmj.com/site/about/guidelines.xhtml

Item description	Explanation	Page in manuscript where item is located	Other*
7. Report any use of components from an existing intervention in the current intervention development process.	Some interventions are developed with components that have been adopted from existing interventions. Clearly identifying components that have been adopted or adapted and acknowledging their original source helps the reader to understand and distinguish between the novel and adopted components of the new intervention.		
8. Report any guiding principles, people or factors that were prioritised when making decisions during the intervention development process.	Reporting any guiding principles that governed the development of the application helps the reader to understand the authors' reasoning behind the decisions that were made. These could include the examples of particular populations who views are being considered when designing the intervention, the modality that is viewed as being most appropriate, design features considered important for the target population, or the potential for the intervention to be scaled up.		
9. Report how stakeholders contributed to the intervention development process.	Potential stakeholders can include patient and community representatives, local and national policy makers, health care providers and those paying for or commissioning health care. Each of these groups may influence the intervention development process in different ways. Specifying how differing groups of stakeholders contributed to the intervention development process helps the reader to understand how stakeholders were involved and the degree of influence they had on the overall process. Further detail on how to integrate stakeholder contributions within intervention reporting are available (19).		
10. Report how the intervention changed in content and format from the start of the intervention development process.	Intervention development is frequently an iterative process. The conclusion of the initial phase of intervention development does not necessarily mean that all uncertainties have been addressed. It is helpful to list remaining uncertainties such as the intervention intensity, mode of delivery, materials, procedures, or type of location that the intervention is most suitable for. This can guide other researchers to potential future areas of research and practitioners about uncertainties relevant to their healthcare context.		
11. Report any changes to interventions required or likely to be required for subgroups.	Specifying any changes that the intervention development team perceive are required for the intervention to be delivered or tailored to specific sub groups enables readers to understand the applicability of the intervention to their target population or context. These changes could include changes to personnel delivering the intervention, to the content of the intervention, or to the mode of delivery of the intervention.		
12. Report important uncertainties at the end of the intervention development process.	Intervention development is frequently an iterative process. The conclusion of the initial phase of intervention development does not necessarily mean that all uncertainties have been addressed. It is helpful to list remaining uncertainties such as the intervention intensity, mode of delivery, materials, procedures, or type of location that the intervention is most suitable for. This can guide other researchers to potential future areas of research and practitioners about uncertainties relevant to their healthcare context.		
13. Report the intervention development process in an open access format.	Unless reports of intervention development are available people considering using an intervention cannot understand the process that was undertaken and make a judgement about its appropriateness to their context. It also limits cumulative learning about intervention development methodology and observed consequences at later evaluation, translation and implementation stages. Reporting intervention development in an open access (Gold or Green) publishing format increases the accessibility and visibility of intervention development research and makes it more likely to be read and used. Potential platforms for open access publication of intervention development include open access journal publications, freely accessible funder reports or a study web-page that details the intervention development process.		

*e.g. if item is reported elsewhere, then the location of this information can be stated here.

For peer review only - http://bmjopen.bmj.com/site/about/guidelines.xhtml

GUIDED – a guideline for reporting for intervention development studies.

Supplementary File 2: Intervention development item examples. Some examples could fit more than one item.

**Item description**	**Examples** (text marked in *italics* highlights aspects directly related to item description)
1. Report the context for which the intervention was developed.	1a "The purpose of this article is to describe the development of a theory-based, culturally relevant intervention focusing on primary (sexual risk reduction) and secondary (Pap smear) prevention of cervical cancer *among Latina immigrants* using intervention mapping (IM)."(1)<br><br>1b "In an effort to bridge this evidence-practice gap, we have developed a behaviour change intervention that aims to increase provision of upper limb repetitive task-oriented training in *stroke rehabilitation*."(2)
2. Report the purpose of the intervention development process.	2a. "The aim of this phase of the project was *to develop a CBTi that could be delivered by* HCAs (non-specialist, relatively low-paid staff)*, after training in basic CBT skills*."(3)
3. Report the target population for the intervention development process.	3a. "The purposes of this study were to use patient-centered, mixed-methods intervention development techniques to develop and refine a computer-based intervention for *drug- using women reporting IPV [intimate partner violence] in the ED [emergency department]…* The intervention was developed in a 5-step process: (1) Initial intervention development based on selected theoretical frameworks; (2) *In-depth interviews with the target population*; (3) Intervention adaptation, with iterative feedback from further interviews; (4) Beta testing and review by an advisory committee of domestic violence advocates; (5) Acceptability and feasibility testing in a small open trial."(4)
4. Report how any published intervention development approach contributed to the development process	4a. *"We applied each of the six 6SQuID steps in relation to five areas of adolescent health-related behaviour* (substance use, sleep, sexual behaviour, physical activity, and eating behaviour), in order to determine how our review findings could further enhance the development of effective, relevant, and sustainable interventions. This involved incorporating the specific neurological changes that happen during adolescence, such as the alterations that occur in the limbic system and pre-frontal cortex, and their influence on areas such as decision-making and reward processing [7]. The steps outlined in Table 1 were therefore considered in relation to adolescent physiology and health-related behaviour, and the results indicate their application to intervention development."(5)<br><br>4b. "*Our approach was based on the key development activities outlined in the MRC framework for the development and evaluation of complex interventions*.[89] There *were four non-sequential activities*, which: A. Identified the existing evidence on interventions to modify explanatory factors for HRQoL (symptoms and ability to function in a normal role and social environment). B. Identified and developed theory to underpin the intervention by convening an expert group to discuss the evidence in the context of expert knowledge and wider theory. C. Modelled processes and outcomes by reconvening the expert group to identify theory-based behaviour change techniques (BCTs), conceptualise the theoretical mechanisms of change in the intervention and identify process and outcome evaluation measures. D. Assessed intervention feasibility and acceptability through interviews with patients who might receive the intervention and health professionals who would deliver it. It was then piloted with a small group of patients"(6)
5. Report how evidence from different sources informed the intervention development process.	5a. "*The three methods used will be realist literature review, secondary analysis of the English Longitudinal Study of Ageing (ELSA), and qualitative focus groups and interviews.* Robust integration of these data will be paramount and figure 1 shows a triangulation protocol detailing how these data will be integrated. *A realist approach will be taken to synthesise and integrate data.*[34] *This theory will be explored with stakeholders to develop an intervention which will be tested and refined in a feasibility trial*."(7)<br><br>5b. "*The [mixed lit review and qualitative interview] findings were discussed with a stakeholder panel to agree the components of an intervention*. Stakeholders included stroke researchers, health professionals and service user representatives from the King's College London Stroke Research Patients and Family Group. Findings were initially presented to stakeholders who were asked to consider in light of the evidence, the types of intervention that might be feasible. The larger group then reconvened for group discussion to co-design an intervention. As a peer support model had been proposed, we scoped this literature to identify likely mechanisms of change that could operate as 'active ingredients' of peer support to improve resilient practices after stroke. We then modelled process and outcomes to develop a theoretically informed intervention to promote resilience after stroke."(7)

For peer review only - http://bmjopen.bmj.com/site/about/guidelines.xhtml

**Item description**	**Examples** (text marked in *italics* highlights aspects directly related to item description)
6. Report how/if published theory informed the intervention development process.	6a “*The content and structure of the video were designed in line with best practice in health psychology and behavior change theory, and recent qualitative research into psychosocial aspects of alcohol use* (Brown & Gregg, 2012; de Visser et al., 2013; Jayne et al., 2010; Livingstone et al., 2011; MacNeela & Bredin, 2011; Szmigin et al., 2011). *The approach involved several different techniques identified in Abraham and Michie’s (2008) taxonomy of behavior change intervention techniques* -providing information about others’ approval; prompting identification of barriers; providing general encouragement; modeling behavior; and providing opportunities for social comparison; providing information about consequences; teaching use of prompts/cues; and planning social support. Employed.”(8)<br><br>6b “*The framework driving the design of this multimedia intervention is the chronic care self-management model (CCM).* The CCM transforms a reactive health care system into one that improves patient outcomes through planning, proven strategies, management, and patient activation [38, 39]. The model results in healthier patients, more satisfied providers, and lower health care utilizations and can be applied to cancer surgical populations [40, 41]. The CCM recognizes six essential elements of high-quality care. These include (1) systems that promote safe, high-quality care; (2) effective, efficient clinical care that includes patient and FCG self-management support; (3) care that is evidence-based and family-centered; (4) efficient and effective care through organized data; (5) care that empowers and prepares patients and FCGs to manage healthcare; and (6) inclusion of community resources [41]. The six essential elements and responsive intervention content are described in Table 1.”(9)
7. Report any use of components from an existing intervention in the current intervention development process.	7a. “I-DECIDE also *builds on the weave project* (Hegarty, O’Doherty, Gunn, Pierce, & Taft, 2008), a randomized, controlled trial that evaluated the effectiveness of a face-to-face counselling intervention in the primary care setting for women experiencing fear of a partner (Hegarty et al., 2013b). The doctors in the intervention group were provided with training in the delivery of a counselling intervention involving woman centered care, active listening, motivational interviewing techniques, and nondirective problem solving to validate and respond to the woman’s experiences and feelings (Hegarty et al.,2013b). *The I-DECIDE intervention aims to translate aspects of the weave counselling intervention, namely, tailored responses and messaging and the use of motivational interviewing and nondirective problem solving tools into an online format.* It can thus be seen as a form of therapeutic intervention, which differentiates I-DECIDE from the other similar websites being developed in the United States, Canada, and New Zealand, which are primarily decision aids.”(10)<br><br>7b. “*Rather than creating an entirely new intervention, it will be developed by adapting a promising seizure management course that already exists to address the needs of PWE visiting the ED.* The course titled ‘Epilepsy awareness and seizure management’ has been offered on a small scale within the third sector to people from a variety of backgrounds, including patients, teachers and care home staff by the UK charity, Epilepsy Society. The society has offered the course since 1998 and given us permission to adapt it. It has not been formally evaluated, but aims to increase participants’ confidence in seizure management. Changes to the existing course will be required since it was developed for delivery to a narrower, fee-paying group. It was not created for delivery within the health service, nor for PWE who visit EDs who can be particularly challenged by epilepsy and may have lower education. 9 17 27 36 37”(11)
8. Report any guiding principles, people or factors that were prioritised when making decisions during the intervention development process.	8a. “…the value of parental participation in pediatric weight loss trials has previously been demonstrated (21) but not reinforced in studies to date. How the involvement of a caregiver influences weight outcomes in minority youth remains an understudied area (9, 22). *It was hypothesized that greater caregiver involvement in treatment would be associated with greater weight loss.”* (12)<br><br>8b. “Offering a DV [domestic violence] intervention in an online format may assist in overcoming some of the barriers encountered in health care settings. Online interventions are being increasingly used as a way of self-managing health conditions, with promising results (Murray, Burns, See Tai, Lai, & Nazareth, 2005). Lintvedt et al. (2013) point out *that an Internet-based intervention is constantly available and accessible from any location. This flexibility allows women to access the intervention at unexpected times when an abusive partner is not present, as opposed to the health care setting where they must schedule an appointment. Delivering an intervention online also allows women to self identify and self-manage without disclosure to a third party.* This may be particularly beneficial for women who are unable or unwilling to disclose the abuse to a health care professional and would not attend a specialized support service because they do not perceive themselves as a ‘DV victim’ (Zink, Elder, Jacobson, & Klostermann, 2004).”(10)

For peer review only

For peer review only - http://bmjopen.bmj.com/site/about/guidelines.xhtml

**Item description**	**Examples** (text marked in *italics* highlights aspects directly related to item description)
9. Report how stakeholders contributed to the intervention development process.	9a. "A key element of the intervention development process was patient and public involvement (PPI)[22]. The REACH-HF programme has an active PPI group consisting of six people from Cornwall with a range of experiences of heart failure and three caregivers of people with heart failure….. *The REACH-HF PPI group helped to design the topic guide for the focus group interviews, they completed and commented on the needs assessment survey and commented on summaries of information from the focus groups*. The group met every 2 months throughout the 12-month needs assessment stage with additional e-mail and postal correspondence between meetings"(13)<br><br>9b. "*We used a qualitative approach in two stages* (Fig. 2), informed by pre-study pilot work with clinicians and interviews with three service users with experience of sharing news of a cancer diagnosis to guide development of the study methodology. *User interviews informed the study in three ways*: (i) the timing of recruitment, (ii) purposive sampling of participants and (iii) interview guide development."(14)
10. Report how the intervention changed in content and format from the start of the intervention development process.	Intervention changes may be substantial (e.g. changing the mode of delivery of an intervention from face to face to online delivery, or relatively more subtle as illustrated below.<br><br>10a. "Our usability evaluation not only assessed the users' ability to perform tasks successfully on the DEF website but also examined the users' broader interaction with the website comprising their thoughts, feelings, and perceptions [103]. The method used to execute this step was a first experience for the research team and was based on regular practice in the field of usability evaluation [103,104]. Detailed activities of this step are described below…… *Following this step, numerous improvements (ie, more than 40) were made, ranging from minor (eg, changing a word that was hard to understand on a page to an easier one) to major (eg, redesigning a whole page).* Here are some of the major improvements made to the website: (1) reduction of the number of pages that must be viewed to complete the enrollment process (ie, 5 pages fewer, from 20 to 15 pages), (2) replacement of the neutral background of videos to backgrounds inspiring PA behavior, (3) redesign and reorganization of the information on the self-monitoring tool page, (4) reduction of the length of the information messages and summary messages by about 50% for almost every tailored motivational session, (5) significant reduction in the number of choices for MI-based multiple choice questions in tailored motivational sessions 3 and 5, (6) redesign of the emails sent to recruit participants, and (7) reduction in the number of times participants have to scroll to almost zero."(15)<br><br>10b "*The text messages developed by different stakeholders in this study were substantially revised by caregivers participating in focus group discussions conducted to test understanding of the messages.* The discussions revealed ambiguities, assumptions and unfamiliar terms used in the messages. Terms like 'AL' and 'reassessment' were replaced with words that were more general and easy to understand. Similarly, a message that required a caregiver to take the child to the hospital immediately if unwell was revised to take into consideration circumstances like lack of transport if a child fell sick at night. *Messages that could have multiple interpretations were identified and revised.* Participants of FGDs preferred personalized SMS messages addressing the caregiver by their specific name. This informed the decision to programme the automated distribution system to send each message with a salutation addressing the caregiver by their name and language of choice. Using their specific names made the caregivers feel that the message was addressing them personally, even though the messages were not tailored to their individual needs." (16)
11. Report any changes to interventions required or likely to be required for subgroups.	12a. "There is the potential to expand this intervention to a wider group of health care professionals involved within cancer care such as oncologists, radiologists, dieticians, speech and language therapists, physiotherapists, occupational therapists and those working in primary care such as GPs, health care assistants and community nurses. If the behavioural diagnosis can be confirmed for these groups then the intervention can be deemed to be feasible for these audiences. There is the potential to adapt this intervention to encourage physical activity in other long-term condition patients by health care professionals. Again, *if the behavioural diagnosis can be confirmed, the structure of the intervention, the selected intervention functions, policy categories, BCTs and modes of delivery could be deemed feasible, with changes made to the intervention content. Further, if the behavioural diagnosis can be confirmed for delivery of physical activity advice by health care professionals to those identified as inactive within the general population, then the intervention structure could again be deemed feasible, with changes made to the intervention content.* In this instance however, the suggested 'teachable moment'[16,20] might not be present and therefore changes might be less likely to occur in the physical activity behaviours of those in receipt of advice."(17)
12. Report important uncertainties at the end of the intervention development process.	Good examples are required for this item.
13. Report the intervention development process in an open access format.	13a. Corder, K., Schiff, A., Kesten, J. M., & van Sluijs, E. M. (2015). Development of a universal approach to increase physical activity among adolescents: the GoActive intervention. BMJ open, 5(8), e008610.(18)<br><br>13b. Free, C., McCarthy, O., French, R. S., Wellings, K., Michie, S., Roberts, I., ... & Edwards, P. (2016). Can text messages increase safer sex behaviours in young people? Intervention development and pilot randomised controlled trial. Health technology assessment (Winchester, England), 20(57), 1.(19)

For peer review only - http://bmjopen.bmj.com/site/about/guidelines.xhtml

References

1. Scarinci IC, Bandura L, Hidalgo B et al. Development of a theory-based (PEN-3 and health belief model), culturally relevant intervention on cervical cancer prevention among Latina immigrants using intervention mapping. *Health Mapping* 2012;13:29-40.
2. Connell LA, McMahon NE, Redfern J et al. Development of a behaviour change intervention to increase upper limb exercise in stroke rehabilitation. *implementation Science* 2015;10:34.
3. Finch TL, Bamford C, Deary V et al. Making sense of a cognitive behavioural therapy intervention for fear of falling: qualitative study of intervention development. *BMC Health Services Research* 2014;14:436.
4. Choo E, Guthrie KM, Mello M et al. I need to hear from women who have 'been there': Developing a woman-focused intervention for drug use and partner violence in the emergency department. *Partner Abuse* 2016;7:193.
5. Pringle J, Doi L, Jindal-Snape D et al. Adolescents and health-related behaviour: using a framework to develop interventions to support positive behaviours. *Pilot and Feasibility Studies* 2018;4:69.
6. Gray NM, Allan JL, Murchie P et al. Developing a community-based intervention to improve quality of life in people with colorectal cancer: a complex intervention development study. *BMJ Open* 2013;3:e002596.
7. Sadler E, Sarre S, Tinker A et al. Developing a novel peer support intervention to promote resilience after stroke. *Health & Social Care in the Community* 2017;25:1590-600.
8. de Visser RO, Graber R, Hart A et al. Using qualitative methods within a mixed-methods approach to developing and evaluating interventions to address harmful alcohol use among young people. *Health Psychology* 2015;34:349.
9. Sun V, Kim JY, Raz DJ et al. Preparing cancer patients and family caregivers for lung surgery: development of a multimedia self-management intervention. *Journal of Cancer Education*. 2018;33:557-63.
10. Tarzia L, Murray E, Humphreys C et al. I-DECIDE: an online intervention drawing on the psychosocial readiness model for women experiencing domestic violence *Women's Health Issues* 2016;26:208-16.
11. Noble A, Marson A, Tudur-Smith C et al. 'Seizure First Aid Training' for people with epilepsy who attend emergency departments, and their family and friends: study protocol for intervention development and a pilot randomised controlled trial. *BMJ Open* 2015;5:e009040.
12. Hartlieb KB, Naar S, Ledgerwood DM et al. Contingency management adapted for African-American adolescents with obesity enhances youth weight loss with caregiver participation: a multiple baseline pilot study. *International Journal of Adolescent Medicine and Health* 2015;29(3).
13. Greaves CJ, Wingham J, Deighan C et al. Optimising self-care support for people with heart failure and their caregivers: development of the Rehabilitation Enablement in Chronic Heart Failure (REACH-HF) intervention using intervention mapping. *Pilot and Feasibility Studies* 2016;2:37.
14. Ewing G, Ngwenya N, Benson J et al. Sharing news of a lung cancer diagnosis with adult family members and friends: a qualitative study to inform a supportive intervention. *Patient Education and Counseling* 2016;99:378-85.
15. Moreau M, Gagnon M-P, Boudreau F. Development of a fully automated, web-based, tailored intervention promoting regular physical activity among insufficiently active adults with type 2 diabetes: integrating the I-change model, self-determination theory, and motivational interviewing components. *JMIR Research Protocols*. 2015;4(1).
16. Githinji S, Jones C, Malinga J, Snow RW, Talisuna A, Zurovac D. Development of a text-messaging intervention to improve treatment adherence and post-treatment review of children with uncomplicated malaria in western Kenya. Malaria journal. 2015;14(1):320.
17. Webb J, Foster J, Poulter E. Increasing the frequency of physical activity very brief advice for cancer patients. Development of an intervention using the behaviour change wheel. *Public Health* 2016;133:45-56.

For peer review only - http://bmjopen.bmj.com/site/about/guidelines.xhtml

For peer review only

18. Corder K, Schiff A, Kesten JM et al. Development of a universal approach to increase physical activity among adolescents: the GoActive intervention. *BMJ Open* 2015;5:e008610.
19. Free C, McCarthy O, French RS, Wellings K, Michie S, Roberts I, et al. Can text messages increase safer sex behaviours in young people? Intervention development and pilot randomised controlled trial. *Health Technology Assessment* 2016;20:1.

For peer review only

For peer review only - http://bmjopen.bmj.com/site/about/guidelines.xhtml

BMJ Open

Guidance for reporting intervention development studies in health research (GUIDED): An evidence-based consensus study.

Journal:	*BMJ Open*
Manuscript ID	bmjopen-2019-033516.R1
Article Type:	Original research
Date Submitted by the Author:	10-Jan-2020
Complete List of Authors:	Duncan, Edward; University of Stirling, NMAHP-RU<br>O'Cathain, Alicia; University of Sheffield, School of Health and Related Research<br>Rousseau, Nikki; University of Stirling<br>Sworn, Katie; University of Sheffield, Health Economics and Decision Science, School of Health and Related Research<br>Turner, Katrina; University of Bristol, School of Social and Community Medicine<br>Yardley, Lucy; University of Southampton, Psychology; University of Bristol, School of Health Sciences<br>Hoddinott, Pat; University of Stirling, Nursing, Midwifery and Allied Health Professional Research Unit
**Primary Subject Heading**:	Health services research
Secondary Subject Heading:	Medical publishing and peer review
Keywords:	Intervention development, Reporting Guidance, Delphi, Consensus

SCHOLARONE™
Manuscripts

I, the Submitting Author has the right to grant and does grant on behalf of all authors of the Work (as defined in the below author licence), an exclusive licence and/or a non-exclusive licence for contributions from authors who are: i) UK Crown employees; ii) where BMJ has agreed a CC-BY licence shall apply, and/or iii) in accordance with the terms applicable for US Federal Government officers or employees acting as part of their official duties; on a worldwide, perpetual, irrevocable, royalty-free basis to BMJ Publishing Group Ltd ("BMJ") its licensees and where the relevant Journal is co-owned by BMJ to the co-owners of the Journal, to publish the Work in this journal and any other BMJ products and to exploit all rights, as set out in our licence.

The Submitting Author accepts and understands that any supply made under these terms is made by BMJ to the Submitting Author unless you are acting as an employee on behalf of your employer or a postgraduate student of an affiliated institution which is paying any applicable article publishing charge ("APC") for Open Access articles. Where the Submitting Author wishes to make the Work available on an Open Access basis (and intends to pay the relevant APC), the terms of reuse of such Open Access shall be governed by a Creative Commons licence – details of these licences and which Creative Commons licence will apply to this Work are set out in our licence referred to above.

Other than as permitted in any relevant BMJ Author's Self Archiving Policies, I confirm this Work has not been accepted for publication elsewhere, is not being considered for publication elsewhere and does not duplicate material already published. I confirm all authors consent to publication of this Work and authorise the granting of this licence.

For peer review only

Guidance for reporting intervention development studies in health research (GUIDED): An evidence-based consensus study.

Authors

Edward Duncan[1]*

Alicia O'Cathain[2]

Nikki Rousseau[2]

Liz Croot[2]

Katie Sworn[2]

Katrina Turner[3]

Lucy Yardley[3,4]

Pat Hoddinott[1]

Author Affiliations:

1. Nursing, Midwifery and Allied Health Professional Research Unit. Faculty of Health Sciences and Sport, University of Stirling, Stirling, Scotland, UK
2. Medical Care Research Unit, School of Health and Related Research, University of Sheffield, Sheffield, UK
3. School of Social and Community Medicine, University of Bristol, Bristol, UK
4. Psychology, University of Southampton, Southampton, UK

***Corresponding author address:**

edward.duncan@stir.ac.uk

NMAHP Research Unit; Innovation Park; University of Stirling; Stirling, Scotland, UK

Word Count = 6,368

For peer review only - http://bmjopen.bmj.com/site/about/guidelines.xhtml

ABSTRACT

Objective: To improve the quality and consistency of intervention development reporting in health research.

Design: A consensus exercise consisting of two simultaneous and identical three round e-Delphi studies (one with experts in intervention development and one with wider stakeholders including funders, journal editors and public involvement members), followed by a consensus workshop. Delphi items were systematically derived from two preceding systematic reviews and a qualitative interview study.

Participants: Intervention developers (n=26) and wider stakeholders (n=18) from the UK, North America and Europe participated in separate e-Delphi studies. Intervention developers (n=13) and wider stakeholders (n=13) participated in a one day consensus workshop.

Results: e-Delphi participants achieved consensus on 15 reporting items. Following feedback from the consensus meeting, the final inclusion and wording of 14 items with description and explanations for each item were agreed. Items focus on context, purpose, target population, approaches, evidence, theory, guiding principles, stakeholder contribution, changes in content or format during the development process, required changes for sub-groups, continuing uncertainties, and open-access publication. They form the GUIDED checklist, which contains a description and explanation of each item, alongside examples of good reporting.

Conclusions: Consensus-based reporting guidance for intervention development in health research is now available for publishers and researchers to use. GUIDED has the potential to lead to greater transparency, and enhance quality and improve learning about intervention development research and practice.

Strengths and Limitations of this Study

- The items were developed through a structured and transparent consensus-based process.
- Parity of opinion was given to intervention developers and wider stakeholders throughout the development of the reporting guidance.
- Despite aiming to secure an international sample this proved difficult. We acknowledge that participants in the study were predominantly based in the Global North and that the perspectives of intervention developers and wider stakeholders from the Global South is absent.

Key words: Intervention Development, Reporting Guidance, Delphi, Consensus

For peer review only

BACKGROUND

The United Kingdom Medical Research Council's (MRC) Framework for the Development and Evaluation of Complex Interventions(1) lists intervention development as the first of a series of interconnected phases. While the MRC complex intervention guidance has stimulated considerable methodological progress in understanding and reporting the latter phases (i.e. feasibility and piloting, evaluation, and implementation), the intervention development phase has remained relatively underdeveloped and without a comprehensive reporting guideline(2). Research funders, researchers, commissioners, practitioners, the public and patients are increasingly interested in understanding and improving the intervention development process.

There are a variety of ways to develop interventions. A review of approaches include partnership (e.g. co-production; co-design), target population centred, evidence and theory-based, implementation-based, efficiency-based, step or phased-based, intervention-specific or a combination of methods(3). Successful intervention development is characterised as being rigorous, scientific and resulting in effective interventions that can be implemented in real world settings(4). However, a key intervention development challenge is the lack of evidence-based quality criteria on which to assess which, if any, approach is superior to another, and in which context.

The reasons why intervention development processes are currently under-reported are unclear. This may be due to research funding priorities or pressure to publish efficacy or effectiveness studies diminishing the priority of publishing intervention development studies. When intervention development studies are published, they are sometimes included as part of a feasibility or pilot study. Consequently, detail about how the intervention was actually developed can be sparse. A more systematic, comprehensive, and transparent approach to intervention development reporting is likely to enhance understanding about the intervention development process. It would help readers to understand the benefits and challenges of different intervention development approaches. It would help researchers select an intervention development approach that is relevant to their context. It would also facilitate future retrospective assessment of how different intervention development approaches can lead to either effective or ineffective interventions that do or do not translate into practice change. Potentially such assessment could provide insights into research waste. While some reporting guidance already exists that relates to intervention development, these are limited in scope. The TIDiER(5) guidelines are extensions to CONSORT for improving the reporting of the completed intervention that results from the intervention development process. The CREDICI guideline for reporting the development and evaluation of Complex Intervention in Healthcare does provide reporting guidance for intervention development, however this is limited to four items as CREDECI 2 also provides guidance on reporting Feasibility and Piloting and Evaluation. To date, there has been no guidance focusing in detail on reporting the whole process of intervention development.

This paper presents GUIDance for the rEporting of intervention Development (GUIDED). GUIDED forms part of a larger MRC funded study to produce guidance on intervention development: the IdentifyiNg and assessing different approaches to DEveloping compleX interventions (INDEX) study(6). It is the first international mixed methods consensus study to focus on reporting guidance solely for intervention development processes. In this paper we report the methods used to develop and gain consensus on the items included in the GUIDED checklist. We present each reporting item with further description and explanation. GUIDED will be of interest to research funders, researchers, journal editors, commissioners, practitioners, the public and patients, who we refer to collectively as "readers".

For peer review only - http://bmjopen.bmj.com/site/about/guidelines.xhtml

METHODS

Design

We published our intent to develop intervention reporting guidance (5 July 2017) in the Equator Network Library (http://www.equator-network.org/library/reporting-guidelines-under-development/reporting-guidelines-under-development-for-other-study-designs/#80). The design of this intervention development consensus study involved conducting two simultaneous and identical e-Delphi studies followed by a consensus workshop. Participants included i) intervention developers and ii) wider stakeholders who were involved in the wider intervention development activities including directors of research funding panels, editors of journals that had published intervention development studies, public and patient involvement members of intervention development studies, and people working in health service implementation(6). By separating intervention developers and wider stakeholders within the e-Delphi process we ensured that the perceptions of both groups were equally reported and their views given equal weight. A subset of the consensus exercise related specifically to the identification of intervention development reporting guidance, reported in this paper. We followed established methods for developing reporting guidance(7) (See Figure 1) and report the e-Delphi guidance in line with current best practice(8). The parallel e-Delphi studies were delivered over three separate rounds. Each round lasted for 4 weeks. Non-responders were emailed a reminder after two weeks. Completion of one round was required to enter the next e-Delphi round. There was space for participants to comment beside each item and explain their responses, or (in Round 1) suggest alternative item wording. Comments were reviewed by the research team, but were not sufficient in number or depth to require analysis using a formal method. No additional items were suggested by participants and no changes were made to the wording of times between rounds. Items were not removed from subsequent rounds, even if they had previously passed the pre-determined threshold.

e-Delphi item generation.

e-Delphi items were generated by triangulating three different data sources: a systematic methods overview of 87 articles, books and websites that identified 23 approaches to intervention development within 8 categories and with 18 actions undertaken across these approaches(3); a systematic review of 87 international primary research articles reporting intervention development processes which describes 10 actions(9); and an analysis of 21 in-depth qualitative interviews with an international sample of intervention developers (N=15) and key stakeholders (N=6)(4, 10). The research team (AO, LY, PH, ED, LC, NR, KS) met regularly to identify the potential reporting guidance items. Members of the research team worked in pairs, and one team of three, to extract potential guidance items from the three data sources. Each pair then presented potential e-Delphi items to the whole team. Each potential item was discussed, refined, and agreed. We grouped items into themes, with one theme entitled "Reporting Guidance", which had 18 items. The full set of e-Delphi items and their ratings have been reported elsewhere(6).

e-Delphi participants.

Invitations were sent to 92 individuals who had undertaken intervention development and/or published a formal approach to intervention development and 80 wider stakeholders. Intervention developers, identified through parallel studies conducted by the research team(3, 9), were invited to participate if they had published at least one intervention development study, or written methodological books or journal articles about intervention development. Wider stakeholders were identified through a web search of Journal editorial boards, funding bodies, and other relevant sources. Wider stakeholders were invited if their role brought them into direct contact with the intervention development process, for example as editors, funding panel members (patient and public involvement members and subject experts), or commissioners. In addition, we convened an

For peer review only - http://bmjopen.bmj.com/site/about/guidelines.xhtml

international expert panel with members from the UK, USA and Europe early in the project to guide the research(6). Approximately one third of invited participants were from countries other than the UK. Members of this expert panel participated in the e-Delphi studies and consensus workshop alongside other participants. Individuals who responded to say they would participate in the e-Delphi were emailed a study information containing a url to an established e-Delphi platform (11) and a unique password to access the study.

Definition of consensus.

Following an online consent process, participants were asked to rate the importance they would give to each potential item, when developing complex interventions to improve health, on a scale of 1 to 5: Not at all important (1); Slightly unimportant (2); Somewhat important (3); Fairly important (4); Very important (5). An additional option of no relevant expertise was provided for each item. In our grant application, we decided that an item would be included within the reporting guidance if at least 70% of participants agreed that an item was fairly important (4) or very important (5) in either e-Delphi group by the end of Round 3. We discussed this with our expert panel prior to undertaking the e-Delphi. Including items that reached the pre-defined threshold in either group meant that equal priority was given to participants that belonged to either the intervention development or wider stakeholder group. A similar approach to methodological guideline reporting development has been used elsewhere.(12)

Consensus meeting.

The results of the e-Delphi studies were discussed by participants (in person or by video link) and eight team members at a one day consensus meeting on 13 March 2018, in London, UK. The meeting began with presentations from the team: an overview of the overall INDEX study, followed by a summary overview of both systematic reviews(3, 9) and qualitative study(4). The results of both e-Delphi studies were then presented and detailed discussions were held on items that had not reached consensus, but which reflected divergence. Consensus meeting participants did not re-rate items but suggested how the wording of some items may have caused confusion. We took this into consideration in our interpretation of the e-Delphi. However, this did not affect any of the reporting items.

Following the consensus meeting.

After the meeting two of the reporting items were merged in the final GUIDED checklist: Item 10 “Report how the intervention changed in content and format from the start of the intervention development process” and a recommendation to “report the reasons for discarding intervention components that were considered”. This was previously a separate item but was considered by the team to be appropriately covered in a single item. No further changes were made to reporting guidance items. We identified examples of previous studies that illustrate use of the reporting items. These examples are almost completely drawn from reviews of previous intervention development literature(4, 9). In one instance, where an example could not be found, an example was created and is identified as a hypothetical example.

*** INSERT FIGURE 1 HERE ***

Patient and public involvement

Patient and public individuals (PPI) were invited a) to an expert panel meeting held at the start of the study, b) to participate in the wider stakeholder eDelphi study, and c) to participate in the consensus meeting. None attended the first meeting, but PPI members did participate in the wider stakeholder eDelphi study and did attend the consensus conference aimed at interpreting the Delphi results. All study participants will be sent copies of journal publications resulting from the study.

For peer review only - http://bmjopen.bmj.com/site/about/guidelines.xhtml

RESULTS

Description of participants

The response rates for each round were as follows: Round 1 Intervention developers (n=34), Wider stakeholders (n=22); Round 2 Intervention developers (n=27), Wider stakeholders (n=18); Round 3 Intervention developers (n=26), Wider stakeholders (n=18). Intervention development participants who completed Round 3 were based in the UK (n=16), mainland Europe (n=5), Ireland (n=4) and USA (n=1). They included people from public health (n=10), applied health research/health services research (n=8), psychology (n=7), nursing (n=6), and allied health professional (n=1) backgrounds. Wider stakeholder participants who completed Round 3 were based in the UK (n=16), mainland Europe (n=1) and USA (n=1). They included chairs or members of funding panels (n=5), editors or editorial board members of journals (n=4), commissioners of services (n=3), public and patient involvement (n=3), and other (n=3) individuals.

The 26 participants of the consensus meeting were based in the UK (n=19), USA (n=3), mainland Europe (n=3), and Ireland (n=1). They were invited due to their varied roles in the intervention development process: intervention developers (n=13), methodologists (n=4), chairs of funding panel (n=3), journal editors (n=3), public and patient representatives (n=1), commissioner (n=1), and other (n=1).

Description of consensus from eDelphi study.

Fifteen of a possible 18 intervention development reporting items reached our a priori threshold for inclusion(6). Table 1 presents all the reporting items included in the e-Delphi; the percentage of responses that scored 4 or 5 for each item and the mode score. The responses to Round 3 of the full Delphi study are available in the full guidance(6).

Table 1. e-Delphi study results for Intervention Development reporting items across each e-Delphi round. Items not reaching the threshold for inclusion in Round 3 are shaded.

Items	Intervention Developers			Wider Stakeholder		
	Mode Score and (% Agreement by Round)					
	Round 1	Round 2	Round 3	Round 1	Round 2	Round 3
Report the purpose of the intervention	5 (97)	5 (96)	5 (100)	5 (94)	5 (100)	5 (100)
Report the target population	5 (97)	5 (96)	5 (100)	5 (100)	5 (100)	5 (100)
Report any use of components from an existing intervention	5 (84)	5 (89)	5 (100)	4 (89)	4 (93)	4 (100)
Report how evidence from different sources informed the intervention development	5 (93)	5 (96)	5 (100)	5 (83)	5 (86)	5 (100)

For peer review only - http://bmjopen.bmj.com/site/about/guidelines.xhtml

Report how stakeholders contributed to the intervention development process	5 (97)	5 (96)	5 (100)	4 (89)	4 (93)	4 (94)
Report important uncertainties at the end of the intervention development process.	5 (87)	5 (93)	5 (100)	5 (83)	5 (86)	5 (78)
Report the context for which the intervention was developed	5 (90)	5 (93)	5 (96)	5 (94)	5 (93)	5 (100)
Report any changes to interventions required or likely to be required for subgroups	5 (90)	5 (89)	5 (96)	5 (83)	4 (93)	4 (83)
Report how any published intervention development approach contributed to the development process	5 (83)	5 (78)	5 (92)	4 (67)	4 (64)	4 (71)
Report how existing published theory informed the intervention development process	5 (87)	5 (89)	5 (92)	4 (89)	5 (93)	4 (94)
Report any guiding principles, people or factors which were prioritised when making decisions	5 (81)	5 (85)	5 (92)	4 (72)	4 (93)	4 (83)
Report how the intervention changed in content and format from the start of the intervention development process*	5 (74)	4 (74)	5 (88)	4 (77)	4 (93)	4 (94)
Report the reasons for discarding intervention components that were considered*	5 (74)	5 (81)	5 (88)	4 (78)	4 (93)	4 (88)
Follow TIDieR guidance when describing the developed intervention	5 (76)	5 (69)	5 (80)	4 (100)	5 (100)	5 (88)
Report the intervention development in an open access format (e.g. open access journal, report chapter, website)	5 (68)	4 (67)	4 (77)	5 (77)	5 (86)	5 (89)
Report the background and contribution of those making decisions about the intervention content, format and delivery	5 (50)	3 (40)	3 (42)	4 (61)	4 (67)	4 (67)
Report the time taken to develop the intervention.	4 (52)	3 (41)	3 (27)	3 (33)	3 (21)	3 (17)
Report who, when, why and where the original idea for developing the intervention came from	3 (45)	3 (30)	3 (27)	5 (50)	4 (64)	4 (67)

*These items were merged into one item (See Item 10 below) following the consensus meeting.

GUIDED intervention development reporting items: Description and explanation

Below, we have ordered the items so that those which are more likely to be considered earlier in the development process are listed first. However, there is no fixed order in which the reporting items

For peer review only

For peer review only - http://bmjopen.bmj.com/site/about/guidelines.xhtml

must be considered. EQUATOR reporting guidance(7) encourages describing and explaining the rationale for each reporting item to help researchers and others to write or appraise reports. We have therefore followed this format, in keeping with other related reporting guidelines(5, 13, 14).

A blank checklist to support the use of GUIDED by authors and reviewers is provided in Supplementary File 1.

Item 1.

Description: Report the context for which the intervention was developed.

Explanation: Understanding the context in which an intervention was developed informs readers about the suitability and transferability of the intervention to the context in which they are considering evaluating, adapting or using the intervention. Context here can include place, organisational and wider socio-political factors that may influence the development and/or delivery of the intervention(15).

Example:

1a "The purpose of this article is to describe the development of a theory-based, culturally relevant intervention focusing on primary (sexual risk reduction) and secondary (Pap smear) prevention of cervical cancer *among Latina immigrants* using intervention mapping (IM)."(16)

1b "In an effort to bridge this evidence-practice gap, we have developed a behaviour change intervention that aims to increase provision of upper limb repetitive task-oriented training in *stroke rehabilitation*."(17)

Item 2.

Description: Report the purpose of the intervention development process.

Explanation: Clearly describing the purpose of the intervention specifies what it sets out to achieve. The purpose may be informed by research priorities, for example those identified in systematic reviews, evidence gaps set out in practice guidance such as The National Institute for Health and Care Excellence or specific prioritisation exercises such as those undertaken with patients and practitioners through the James Lind Alliance.

Example:

2a. "The aim of this phase of the project was *to develop a CBTi that could be delivered by* HCAs (non-specialist, relatively low-paid staff)*, after training in basic CBT skills*."(18)

Item 3.

Description: Report the target population for the intervention development process.

Explanation: The target population is the population that will potentially benefit from the intervention – this may include patients, clinicians, and/or members of the public. If the target population is clearly described then readers will be able to understand the relevance of the intervention to their own research or practice. Health inequalities, gender and ethnicity are features of the target population that may be relevant to intervention development processes.

Example:

3a. "The purposes of this study were to use patient-centered, mixed-methods intervention development techniques to develop and refine a computer-based intervention for *drug- using women reporting IPV [intimate partner violence] in the ED [emergency department]…* The intervention was developed in a 5-step process: (1) Initial intervention development based on selected theoretical frameworks; (2) *In-depth interviews with the target population*; (3) Intervention adaptation, with iterative feedback from further interviews; (4) Beta testing and review by an

For peer review only - http://bmjopen.bmj.com/site/about/guidelines.xhtml

advisory committee of domestic violence advocates; (5) Acceptability and feasibility testing in a small open trial."(19)

Item 4.

Description: Report how any published intervention development approach contributed to the development process.

Explanation: Many formal intervention development approaches exist and are used to guide the intervention development process (e.g. 6Squid(20) or The Person Based Approach to Intervention Development(21)). Where a formal intervention development approach is used, it is helpful to describe the process that was followed, including any deviations. More general approaches to intervention development also exist and have been categorised as follows(3):- Target Population-centred intervention development; evidence and theory-based intervention development; partnership intervention development; implementation-based intervention development; efficacy-based intervention development; step or phased-based intervention development; and intervention-specific intervention development(3). These approaches do not always have specific guidance that describe their use. Nevertheless, it is helpful to give a rich description of how any published approach was operationalised.

Example:

4a. *"We applied each of the six 6SQuID steps in relation to five areas of adolescent health-related behaviour* (substance use, sleep, sexual behaviour, physical activity, and eating behaviour), in order to determine how our review findings could further enhance the development of effective, relevant, and sustainable interventions. This involved incorporating the specific neurological changes that happen during adolescence, such as the alterations that occur in the limbic system and pre-frontal cortex, and their influence on areas such as decision-making and reward processing [7]. The steps outlined in Table 1 were therefore considered in relation to adolescent physiology and health-related behaviour, and the results indicate their application to intervention development."(22)

4b. "*Our approach was based on the key development activities outlined in the MRC framework for the development and evaluation of complex interventions.*[89] There *were four non-sequential activities*, which: A. Identified the existing evidence on interventions to modify explanatory factors for HRQoL (symptoms and ability to function in a normal role and social environment). B. Identified and developed theory to underpin the intervention by convening an expert group to discuss the evidence in the context of expert knowledge and wider theory. C. Modelled processes and outcomes by reconvening the expert group to identify theory-based behaviour change techniques (BCTs), conceptualise the theoretical mechanisms of change in the intervention and identify process and outcome evaluation measures. D. Assessed intervention feasibility and acceptability through interviews with patients who might receive the intervention and health professionals who would deliver it. It was then piloted with a small group of patients."(23)

Item 5.

Description: Report how evidence from different sources informed the intervention development process.

Explanation: Intervention development is often based on published evidence and/or primary data that has been collected to inform the intervention development process. It is useful to describe and reference all forms of evidence and data that have informed the development of the intervention

For peer review only - http://bmjopen.bmj.com/site/about/guidelines.xhtml

because evidence bases can change rapidly, and to explain the manner in which the evidence and/or data was used. Understanding what evidence was and was not available at the time of intervention development can help readers to assess transferability to their current situation.

Example:

5a. “*The three methods used will be realist literature review, secondary analysis of the English Longitudinal Study of Ageing (ELSA), and qualitative focus groups and interviews…. A realist approach will be taken to synthesise and integrate data.*[34] *This theory will be explored with stakeholders to develop an intervention which will be tested and refined in a feasibility trial.*”(24)

5b. “*The [mixed lit review and qualitative interview] findings were discussed with a stakeholder panel to agree the components of an intervention*. Stakeholders included stroke researchers, health professionals and service user representatives from the King’s College London Stroke Research Patients and Family Group. Findings were initially presented to stakeholders who were asked to consider in light of the evidence, the types of intervention that might be feasible. The larger group then reconvened for group discussion to co-design an intervention. As a peer support model had been proposed, we scoped this literature to identify likely mechanisms of change that could operate as ‘active ingredients’ of peer support to improve resilient practices after stroke. We then modelled process and outcomes to develop a theoretically informed intervention to promote resilience after stroke.”(24)

Item 6.

Description: Report how/if existing published theory informed the intervention development process.

Explanation: Reporting whether and how theory informed the intervention development process aids the reader’s understanding of the theoretical rationale that underpins the intervention. Though not mentioned in the e-Delphi or consensus meeting, it became increasingly apparent through the development of our guidance that this item could relate to either existing published theory or programme theory.

Example:

6a “*The content and structure of the video were designed in line with best practice in health psychology and behavior change theory, and recent qualitative research into psychosocial aspects of alcohol use* (Brown & Gregg, 2012; de Visser et al., 2013; Jayne et al., 2010; Livingstone et al., 2011; MacNeela & Bredin, 2011; Szmigin et al., 2011). *The approach involved several different techniques identified in Abraham and Michie’s (2008) taxonomy of behavior change intervention techniques* - providing information about others’ approval; prompting identification of barriers; providing general encouragement; modeling behavior; and providing opportunities for social comparison; providing information about consequences; teaching use of prompts/cues; and planning social support. Employed.”(25)

6b “*The framework driving the design of this multimedia intervention is the chronic care self-management model (CCM).* The CCM transforms a reactive health care system into one that improves patient outcomes through planning, proven strategies, management, and patient activation [38, 39]. The model results in healthier patients, more satisfied providers, and lower health care utilizations and can be applied to cancer surgical populations [40, 41]. The CCM recognizes six essential elements of high-quality care. These include (1) systems that promote safe, high-quality care; (2) effective, efficient clinical care that includes patient and FCG self-management support; (3) care that is evidence-based and family-centered; (4) efficient and effective care through organized data; (5) care that empowers and prepares patients and FCGs to manage healthcare; and

For peer review only - http://bmjopen.bmj.com/site/about/guidelines.xhtml

(6) inclusion of community resources [41]. The six essential elements and responsive intervention content are described in Table 1."(26)

Item 7.

Description: Report any use of components from an existing intervention in the current intervention development process.

Explanation: Some interventions are developed with components that have been adopted from existing interventions. Clearly identifying components that have been adopted or adapted and acknowledging their original source helps the reader to understand and distinguish between the novel and adopted components of the new intervention.

Example:

7a. "I-DECIDE also *builds on the weave project* (Hegarty, O'Doherty, Gunn, Pierce, & Taft, 2008), a randomized, controlled trial that evaluated the effectiveness of a face-to-face counselling intervention in the primary care setting for women experiencing fear of a partner (Hegarty et al., 2013b). The doctors in the intervention group were provided with training in the delivery of a counselling intervention involving woman centered care, active listening, motivational interviewing techniques, and nondirective problem solving to validate and respond to the woman's experiences and feelings (Hegarty et al.,2013b). *The I-DECIDE intervention aims to translate aspects of the weave counselling intervention, namely, tailored responses and messaging and the use of motivational interviewing and nondirective problem solving tools into an online format.* It can thus be seen as a form of therapeutic intervention, which differentiates I-DECIDE from the other similar websites being developed in the United States, Canada, and New Zealand, which are primarily decision aids."(27)

7b. "*Rather than creating an entirely new intervention, it will be developed by adapting a promising seizure management course that already exists to address the needs of PWE visiting the ED*. The course titled 'Epilepsy awareness and seizure management' has been offered on a small scale within the third sector to people from a variety of backgrounds, including patients, teachers and care home staff by the UK charity, Epilepsy Society. The society has offered the course since 1998 and given us permission to adapt it. It has not been formally evaluated, but aims to increase participants' confidence in seizure management. Changes to the existing course will be required since it was developed for delivery to a narrower, fee-paying group. It was not created for delivery within the health service, nor for PWE who visit EDs who can be particularly challenged by epilepsy and may have lower education. 9 17 27 36 37."(28)

Item 8.

Description: Report any guiding principles, people or factors that were prioritised when making decisions during the intervention development process.

Explanation: Reporting any guiding principles that governed the development of the intervention will help the reader to understand the authors' reasoning behind the decisions that were made. Guiding principles specify the core objectives and features of the desired intervention(29). These could include prioritising patient preferences over clinician preferences, providing an engaging experience for patients, minimizing the cost of delivering the intervention, or maximising the potential for the intervention to be scaled up.

Example:

8a. "…the value of parental participation in pediatric weight loss trials has previously been demonstrated (21) but not reinforced in studies to date. How the involvement of a caregiver influences weight outcomes in minority youth remains an understudied area (9, 22). *It was*

For peer review only - http://bmjopen.bmj.com/site/about/guidelines.xhtml

hypothesized that greater caregiver involvement in treatment would be associated with greater weight loss." (30)

8b. "Offering a DV [domestic violence] intervention in an online format may assist in overcoming some of the barriers encountered in health care settings. Online interventions are being increasingly used as a way of self-managing health conditions, with promising results (Murray, Burns, See Tai, Lai, & Nazareth, 2005). Lintvedt et al. (2013) point out *that an Internet-based intervention is constantly available and accessible from any location. This flexibility allows women to access the intervention at unexpected times when an abusive partner is not present, as opposed to the health care setting where they must schedule an appointment. Delivering an intervention online also allows women to self identify and self-manage without disclosure to a third party.* This may be particularly beneficial for women who are unable or unwilling to disclose the abuse to a health care professional and would not attend a specialized support service because they do not perceive themselves as a 'DV victim' (Zink, Elder, Jacobson, & Klostermann, 2004)."(27)

Item 9.

Description: Report how stakeholders contributed to the intervention development process.

Explanation: Potential stakeholders can include patient and community representatives, local and national policy makers, health care providers and those paying for or commissioning health care. Each of these groups may influence the intervention development process in different ways. Specifying how differing groups of stakeholders contributed to the intervention development process helps the reader to understand how stakeholders were involved and the degree of influence they had on the overall process. Further detail on how to integrate stakeholder contributions within intervention reporting are available(31).

Example:

9a. "A key element of the intervention development process was patient and public involvement (PPI)[22]. The REACH-HF programme has an active PPI group consisting of six people from Cornwall with a range of experiences of heart failure and three caregivers of people with heart failure….. *The REACH-HF PPI group helped to design the topic guide for the focus group interviews, they completed and commented on the needs assessment survey and commented on summaries of information from the focus groups*. The group met every 2 months throughout the 12-month needs assessment stage with additional e-mail and postal correspondence between meetings."(32)

9b. "*We used a qualitative approach in two stages* (Fig. 2), informed by pre-study pilot work with clinicians and interviews with three service users with experience of sharing news of a cancer diagnosis to guide development of the study methodology. *User interviews informed the study in three ways*: (i) the timing of recruitment, (ii) purposive sampling of participants and (iii) interview guide development."(33)

Item 10.

Description: Report how the intervention changed in content and format from the start of the intervention development process.

Explanation: Due to the iterative nature of intervention development, the intervention that is defined at the end of the development process can often be quite different to the one that was initially planned. Describing these changes and their rationale enhances understanding and enables other intervention developers to learn from this experience. For example, it may be that some intervention components were considered but ultimately discarded due to complexity or expense of delivery.

For peer review only - http://bmjopen.bmj.com/site/about/guidelines.xhtml

Example:

Intervention changes may be substantial (e.g. changing the mode of delivery of an intervention from face to face to online delivery, or relatively more subtle as illustrated below.

10a. “Our usability evaluation not only assessed the users’ ability to perform tasks successfully on the DEF website but also examined the users’ broader interaction with the website comprising their thoughts, feelings, and perceptions [103]. The method used to execute this step was a first experience for the research team and was based on regular practice in the field of usability evaluation [103,104]. Detailed activities of this step are described below…… *Following this step, numerous improvements (ie, more than 40) were made, ranging from minor (eg, changing a word that was hard to understand on a page to an easier one) to major (eg, redesigning a whole page).* Here are some of the major improvements made to the website: (1) reduction of the number of pages that must be viewed to complete the enrollment process (ie, 5 pages fewer, from 20 to 15 pages), (2) replacement of the neutral background of videos to backgrounds inspiring PA behavior, (3) redesign and reorganization of the information on the self-monitoring tool page, (4) reduction of the length of the information messages and summary messages by about 50% for almost every tailored motivational session, (5) significant reduction in the number of choices for MI-based multiple choice questions in tailored motivational sessions 3 and 5, (6) redesign of the emails sent to recruit participants, and (7) reduction in the number of times participants have to scroll to almost zero.”(34)

10b “*The text messages developed by different stakeholders in this study were substantially revised by caregivers participating in focus group discussions conducted to test understanding of the messages.* The discussions revealed ambiguities, assumptions and unfamiliar terms used in the messages. Terms like ‘AL’ and ‘reassessment’ were replaced with words that were more general and easy to understand. Similarly, a message that required a caregiver to take the child to the hospital immediately if unwell was revised to take into consideration circumstances like lack of transport if a child fell sick at night. *Messages that could have multiple interpretations were identified and revised.* Participants of FGDs preferred personalized SMS messages addressing the caregiver by their specific name. This informed the decision to programme the automated distribution system to send each message with a salutation addressing the caregiver by their name and language of choice. Using their specific names made the caregivers feel that the message was addressing them personally, even though the messages were not tailored to their individual needs.” (35)

Item 11.

Description: Report any changes to interventions required or likely to be required for subgroups.

Explanation: Specifying any changes that the intervention development team perceive are required for the intervention to be delivered or tailored to specific sub groups enables readers to understand the applicability of the intervention to their target population or context. These changes could include changes to personnel delivering the intervention, to the content of the intervention, or to the mode of delivery of the intervention.

Example:

11a. “There is the potential to expand this intervention to a wider group of health care professionals involved within cancer care such as oncologists, radiologists, dieticians, speech and language therapists, physiotherapists, occupational therapists and those working in primary care such as GPs, health care assistants and community nurses. If the behavioural diagnosis can be confirmed for these groups then the intervention can be deemed to be feasible for these audiences. There is the potential to adapt this intervention to encourage physical activity in other long-term condition patients by health care professionals. Again, *if the behavioural diagnosis can be confirmed, the structure of the intervention, the selected intervention functions, policy categories, BCTs and modes*

For peer review only - http://bmjopen.bmj.com/site/about/guidelines.xhtml

of delivery could be deemed feasible, with changes made to the intervention content. Further, if the behavioural diagnosis can be confirmed for delivery of physical activity advice by health care professionals to those identified as inactive within the general population, then the intervention structure could again be deemed feasible, with changes made to the intervention content. In this instance however, the suggested 'teachable moment'[16,20] might not be present and therefore changes might be less likely to occur in the physical activity behaviours of those in receipt of advice."(36)

Item 12.

Description: Report important uncertainties at the end of the intervention development process.

Explanation: Intervention development is frequently an iterative process. The conclusion of the initial phase of intervention development does not necessarily mean that all uncertainties have been addressed. It is helpful to list remaining uncertainties such as the intervention intensity, mode of delivery, materials, procedures, or type of location that the intervention is most suitable for. This can guide other researchers to potential future areas of research and practitioners about uncertainties relevant to their healthcare context.

Example:

No good quality illustration of this item could be found in the literature. The following hypothetical example is offered to illustrate how this item could be reported.

12a. At the conclusion of the intervention development process, we were able to describe the rationale and theoretical basis for the intervention in detail. We were also able to list the materials that are required to deliver the intervention and the training materials that are required to train staff in its use. The intervention should be delivered face to face and provided individually. *While it may be possible for the number of intervention sessions to be personalised depending on the severity of participant's condition, we did not investigate participant or carer acceptability of a variable intervention intensity delivery approach.*

Item 13.

Description: Follow TIDieR guidance when describing the developed intervention.

Explanation: Interventions have been poorly reported for a number of years. In response to this, internationally recognized guidance has been published to support the high quality reporting of health care? interventions(5)and public health interventions(13). This guidance should therefore be followed when describing a developed intervention.

Example:

13a. "*Using the TIDieR framework [21], we created abroad outline of the intervention* that included the content delivered, to whom and by whom, why, by what mode of delivery and how often. Data from all three datasources were used."(37)

13b. "*The 'Template for Intervention Description and Replication' (TIDieR) checklist [16] has been used to structure the description of this intervention.* The 12 checklist items are displayed in Table A. (available online), alongside how each has been interpreted for this intervention description."(38)

Item 14.

Description: Report the intervention development process in an open access format.

Explanation: Unless reports of intervention development are available people considering using an intervention cannot understand the process that was undertaken and make a judgement about its appropriateness to their context. It also limits cumulative learning about intervention development methodology and observed consequences at later evaluation, translation and implementation

For peer review only - http://bmjopen.bmj.com/site/about/guidelines.xhtml

stages. Reporting intervention development in an open access (Gold or Green) publishing format increases the accessibility and visibility of intervention development research and makes it more likely to be read and used. Potential platforms for open access publication of intervention development include open access journal publications, freely accessible funder reports or a study web-page that details the intervention development process.

Example:

14a. Corder, K., Schiff, A., Kesten, J. M., & van Sluijs, E. M. (2015). Development of a universal approach to increase physical activity among adolescents: the GoActive intervention. BMJ open, 5(8), e008610.(39)

14b. Free, C., McCarthy, O., French, R. S., Wellings, K., Michie, S., Roberts, I., ... & Edwards, P. (2016). Can text messages increase safer sex behaviours in young people? Intervention development and pilot randomised controlled trial. Health technology assessment, 20(57), 1.(40)

DISCUSSION

Intervention development is a vital component of the MRC Developing and evaluating complex interventions guidance. This study presents a mixed method international consensus study to produce detailed reporting guidance for the intervention development phase of the MRC Complex Intervention Guidance. The GUIDED checklist provides a list of 14 intervention development items each with an accompanying explanation for why it is important to include this information in publications and outputs that describe the intervention development process. The GUIDED checklist was developed in collaboration with a range of stakeholders, each of whom contributed a range of expertise and perspectives on the intervention development process. Despite efforts to include participation from a global audience, the majority of participants come from within the UK. Among developers, we had a good response from European countries but a poorer response from the rest of the world. Among wider stakeholders, the response was poor from all outside the UK. It seems likely that the study was more relevant to developers and that developers were also more likely to know of the study team, which may have influenced participation. To maximise response, any similar research in the future may benefit from a preliminary email endorsement from an influential person based in the same geographical region as the intended participant.

The GUIDED reporting checklist and its associated item descriptions have been systematically developed to support readers to understand key aspects of specific intervention development studies. Adhering to the GUIDED item checklist across the variety of formats in which intervention development publications already occur should improve the quality, transparency and consistency of intervention development reporting.

What gap does GUIDED fill?

Good quality effectiveness studies with detailed guidance on intervention description are necessary(5, 13). GUIDED is offered as complementary reporting guidance to detail the intervention development process. Presenting intervention development studies in line with GUIDED recommendations reported in this paper will enable commissioners and practitioners to understand the context and methods that were used to develop the intervention to help them make judgements about the quality and relevance of the intervention. This information will be useful in guiding their decisions about whether to evaluate or implement an intervention within their specific context. Finally, high quality and transparent reporting of intervention development in line with GUIDED recommendations will enable methodological lessons to be learnt and incorporated into future intervention development studies. We therefore recommend that authors follow GUIDED when reporting intervention development studies, and journal editors and research funders endorse the use of GUIDED within any publications that report intervention development studies. The GUIDED

For peer review only - http://bmjopen.bmj.com/site/about/guidelines.xhtml

checklist will be placed on the EQUATOR network website and we request that Journals provide links to the EQUATOR site and signpost potential authors to this guidance where appropriate.

How does GUIDED fit with other reporting guidance?

GUIDED provides a more comprehensive description than previous guidance(41) of what should be reported when publishing intervention development studies. GUIDED complements and can easily be integrated or signposted to within other reporting guidance. Papers that are written to describe interventions should follow existing guidance(5, 13) and signpost readers to where they can read about the intervention development process, reported in line with GUIDED recommendations, so they can judge the appropriateness of the intervention development process. Where randomised controlled trials are being reported using CONSORT guidance(14) then authors could signpost (for example in reporting CONSORT Statement 5: Intervention) to where a GUIDED description of intervention development has been reported. Where patients and the public contributed to intervention development, the GRIPP 2 guidelines can be used(31).

CONCLUSION

The GUIDED checklist and reporting guidance has been developed by following internationally recognised methods for developing reporting guidance(7), with items based on extensive primary(4, 42) and secondary(3, 9) research to enable greater transparency and quality of reporting development of complex interventions. The GUIDED checklist and guidance provide a clear and structured basis for the reporting of intervention development studies in a range of formats. It has the potential to facilitate learning about how early intervention development decisions impact across the life history of an intervention: through feasibility and efficacy testing, cost-effectiveness evaluations and translation into health care practice change.

Acknowledgements

We would like to thank all the eDelphi study and consensus conference participants, and members of our expert panel.

Contributors

AOC and PH led the development of the overall intervention development guidance. ED led the consensus exercise working with NR. ED wrote the first draft of the article and integrated contributions from the author group into subsequent drafts. All authors contributed to the design and content of the reporting guidance and approved subsequent drafts of the paper (AOC, PH, LY, LC, NR, KMT, ED, KS).

Funding MRC-NIHR Methodology Research Panel (MR/N015339/1). Funders had no influence on the guidance presented here. The authors were fully independent of the funders.

Competing interests None declared.

Patient consent for publication Not required.

Provenance and peer review Not commissioned; externally peer reviewed.

Data sharing statement De-identified participant responses to the eDelphi study are available from edward.duncan@stir.ac.uk upon reasonable request.

Open access This is an open access article distributed in accordance with the Creative Commons Attribution 4.0 Unported (CC BY 4.0) license, which permits others to copy, redistribute, remix, transform and build upon this work for any purpose, provided the original work is properly cited, a link to the licence is given, and indication of whether changes were made. See: https://creativecommons.org/licenses/by/4.0/.

For peer review only - http://bmjopen.bmj.com/site/about/guidelines.xhtml

For peer review only

References

1. Craig P, Dieppe P, Macintyre S, et al. Developing and evaluating complex interventions: the new Medical Research Council guidance. *British Medical Journal*. 2008;337:a1655.

2. Hoddinott P. A new era for intervention development studies. *Pilot and Feasibility Studies*. 2015(1):36.

3. O'Cathain A, Croot L, Sworn K, et al. Taxonomy of approaches to developing interventions to improve health: a systematic methods overview. *Pilot and Feasibility Studies*. 2019(1):41.

4. Turner KM, Rousseau N, Croot L, et al. Understanding successful development of complex health and healthcare interventions and its drivers from the perspective of developers and wider stakeholders: an international qualitative interview study. *BMJ Open*. 2019;9(5):e028756.

5. Hoffmann TC, Glasziou PP, Boutron I, et al. Better reporting of interventions: template for intervention description and replication (TIDieR) checklist and guide. *British Medical Journal*. 2014;348:g1687.

6. O'Cathain A, Croot L, Duncan E, et al. Guidance on how to develop complex interventions to improve health and healthcare. *BMJ Open*. 2019;9(8):e029954.

7. Moher D, Schulz KF, Simera I, et al. Guidance for developers of health research reporting guidelines. *PLOS Medicine*. 2010;7(2):e1000217.

8. Hasson F, Keeney S, McKenna H. Research guidelines for the Delphi survey technique. *Journal of Advanced Nursing*. 2000;32(4):1008-15.

9. Croot L, O'Cathain A, Sworn K, et al. Developing interventions to improve health: a systematic mapping review of international practice between 2015 and 2016. *Pilot and Feasibility Studies*. 2019;5(1):127.

10. Rousseau N, Turner KM, Duncan E, et al. Attending to design when developing complex health interventions: A qualitative interview study with intervention developers and associated stakeholders. *PLOS ONE*. 2019;14(10).

11. Duncan E, K S. The Stirling eDelphi Platform Stirling, UK: University of Stirling; 2015 [Available from: http://delphi.stir.ac.uk/overview.pdf.

12. France EF, Cunningham M, Ring N, et al. Improving reporting of meta-ethnography: the eMERGe reporting guidance. *BMC Medical Research Methodology*. 2019;19(1):25.

13. Armstrong R, Campbell M, Craig P, et al. Reporting guidelines for population health and policy interventions: TIDieR-PHP. The Lancet. 2015;386:S19.

14. Eldridge SM, Chan CL, Campbell MJ, Bond CM, Hopewell S, Thabane L, et al. CONSORT 2010 statement: extension to randomised pilot and feasibility trials. *Pilot and Feasibility Studies*. 2016;2(1):64.

15. Craig P, Di Ruggiero E, Frolich KL, et al. Taking account of context in population health intervention research: guidance for producers, users and funders of research. 2018;Apr *NIHR Journals Library*

16. Scarinci IC, Bandura L, Hidalgo B, et al. Development of a theory-based (PEN-3 and health belief model), culturally relevant intervention on cervical cancer prevention among Latina immigrants using intervention mapping. *Health Promotion Practice*. 2012;13(1):29-40.

17. Connell LA, McMahon NE, Redfern J, et al. Development of a behaviour change intervention to increase upper limb exercise in stroke rehabilitation. *Implementation Science*. 2015;10(1):34.

For peer review only - http://bmjopen.bmj.com/site/about/guidelines.xhtml

18. Finch TL, Bamford C, Deary V, et al. Making sense of a cognitive behavioural therapy intervention for fear of falling: qualitative study of intervention development. *BMC Health Services Research*. 2014;14(1):436.

19. Choo E, Guthrie KM, Mello M, et al. I need to hear from women who have 'been there': Developing a woman-focused intervention for drug use and partner violence in the emergency department. *Partner Abuse*. 2016;7(2):193.

20. Wight D, Wimbush E, Jepson R, et al. Six steps in quality intervention development (6SQuID). *Journal of Epidemiology and Community Health*. 2016;70(5):520-5.

21. Yardley L, Morrison L, Bradbury K, et al. The person-based approach to intervention development: application to digital health-related behavior change interventions. J*ournal of Medical Internet Research*. 2015;17(1):e30.

22. Pringle J, Doi L, Jindal-Snape D, et al. Adolescents and health-related behaviour: using a framework to develop interventions to support positive behaviours. *Pilot and Feasibility Studies*. 2018;4(1):69.

23. Gray NM, Allan JL, Murchie P, et al. Developing a community-based intervention to improve quality of life in people with colorectal cancer: a complex intervention development study. *BMJ Open*. 2013;3(4):e002596.

24. Sadler E, Sarre S, Tinker A, et al. Developing a novel peer support intervention to promote resilience after stroke. *Health & Social Care in the Community*. 2017;25(5):1590-600.

25. de Visser RO, Graber R, Hart A, et al. Using qualitative methods within a mixed-methods approach to developing and evaluating interventions to address harmful alcohol use among young people. *Health Psychology*. 2015;34(4):349.

26. Sun V, Kim JY, Raz DJ, et al. Preparing cancer patients and family caregivers for lung surgery: development of a multimedia self-management intervention. *Journal of Cancer Education*. 2018;33(3):557-63.

27. Tarzia L, Murray E, Humphreys C, et al. I-DECIDE: an online intervention drawing on the psychosocial readiness model for women experiencing domestic violence. *Women's Health Issues*. 2016;26(2):208-16.

28. Noble A, Marson A, Tudur-Smith C, et al. 'Seizure First Aid Training'for people with epilepsy who attend emergency departments, and their family and friends: study protocol for intervention development and a pilot randomised controlled trial. *BMJ Open*. 2015;5(7):e009040.

29. Morrison L, Muller I, Yardley L, et al. The person-based approach to planning, optimising, evaluating and implementing behavioural health interventions. *The European Health Psychologist*. 2018;20(3):464-9.

30. Hartlieb KB, Naar S, Ledgerwood DM, et al. Contingency management adapted for African-American adolescents with obesity enhances youth weight loss with caregiver participation: a multiple baseline pilot study. *International Journal of Adolescent Medicine and Health*. 2015;29(3).

31. Staniszewska S, Brett J, Simera I, et al. GRIPP2 reporting checklists: tools to improve reporting of patient and public involvement in research. *Research Involvement and Engagement*. 2017;3(1):13.

32. Greaves CJ, Wingham J, Deighan C, et al. Optimising self-care support for people with heart failure and their caregivers: development of the Rehabilitation Enablement in Chronic Heart Failure (REACH-HF) intervention using intervention mapping. *Pilot and Feasibility Studies*. 2016;2(1):37.

For peer review only - http://bmjopen.bmj.com/site/about/guidelines.xhtml

33. Ewing G, Ngwenya N, Benson J, et al. Sharing news of a lung cancer diagnosis with adult family members and friends: a qualitative study to inform a supportive intervention. *Patient Education and Counseling*. 2016;99(3):378-85.

34. Moreau M, Gagnon M-P, Boudreau F. Development of a fully automated, web-based, tailored intervention promoting regular physical activity among insufficiently active adults with type 2 diabetes: integrating the I-change model, self-determination theory, and motivational interviewing components. *JMIR Research Protocols*. 2015;4(1).

35. Githinji S, Jones C, Malinga J, et al. Development of a text-messaging intervention to improve treatment adherence and post-treatment review of children with uncomplicated malaria in western Kenya. *Malaria Journal*. 2015;14(1):320.

36. Webb J, Foster J, Poulter E. Increasing the frequency of physical activity very brief advice for cancer patients. Development of an intervention using the behaviour change wheel. *Public Health*. 2016;133:45-56.

37. Steinmo S, Fuller C, Stone SP, et al. Characterising an implementation intervention in terms of behaviour change techniques and theory: the 'Sepsis Six'clinical care bundle. *Implementation Science*. 2015;10(1):111.

38. Cunningham P, Turton AJ, Van Wijck F, et al. Task-specific reach-to-grasp training after stroke: development and description of a home-based intervention. *Clinical Rehabilitation*. 2016;30(8):731-40.

39. Corder K, Schiff A, Kesten JM, et al. Development of a universal approach to increase physical activity among adolescents: the GoActive intervention. *BMJ Open*. 2015;5(8):e008610.

40. Free C, McCarthy O, French RS, et al. Can text messages increase safer sex behaviours in young people? Intervention development and pilot randomised controlled trial. *Health Technology Assessment* 2016;20(57):1.

41. Möhler R, Köpke S, Meyer G. Criteria for Reporting the Development and Evaluation of Complex Interventions in healthcare: revised guideline (CReDECI 2). *Trials*. 2015;16(1):204.

42. Rousseau N, Turner KM, Duncan E, et al. Attending to design when developing complex health interventions: A qualitative interview study with intervention developers and associated stakeholders. *PLOS ONE*. 2019;14(10).

Figure and Table Captions.

Figure 1: Methods used to develop reporting guidance for intervention development studies in health research.

Fig 2: See - Ewing G, Ngwenya N, Benson J, et al. Sharing news of a lung cancer diagnosis with adult family members and friends: a qualitative study to inform a supportive intervention. *Patient Education and Counseling*. 2016;99(3):378-85.

Table A: See **-** Cunningham P, Turton AJ, Van Wijck F, et al. Task-specific reach-to-grasp training after stroke: development and description of a home-based intervention. *Clinical Rehabilitation*. 2016;30(8):731-40.

For peer review only - http://bmjopen.bmj.com/site/about/guidelines.xhtml

For peer review only

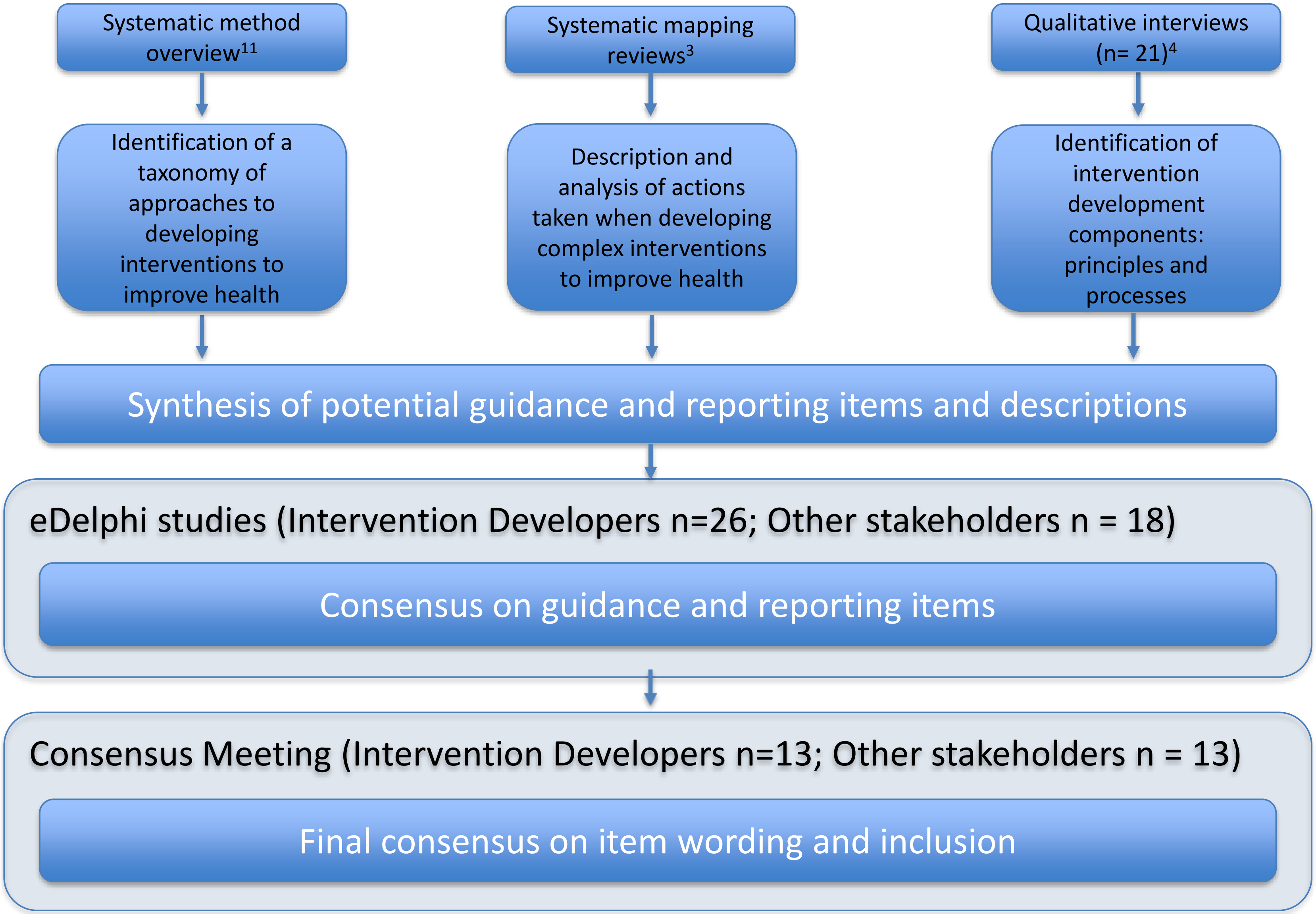
Systematic method overview[11]
Systematic mapping reviews[3]
Qualitative interviews (n= 21)[4]
Identification of a taxonomy of approaches to developing interventions to improve health
Description and analysis of actions taken when developing complex interventions to improve health
Identification of intervention development components: principles and processes
Synthesis of potential guidance and reporting items and descriptions
eDelphi studies (Intervention Developers n=26; Other stakeholders n = 18)
Consensus on guidance and reporting items
Consensus Meeting (Intervention Developers n=13; Other stakeholders n = 13)
Final consensus on item wording and inclusion

GUIDED – a guideline for reporting for intervention development studies.

Supplementary File 1: Blank Checklist

Item description	Explanation	Page in manuscript where item is located	Other*
1. Report the context for which the intervention was developed.	Understanding the context in which an intervention was developed informs readers about the suitability and transferability of the intervention to the context in which they are considering evaluating, adapting or using the intervention. Context here can include place, organisational and wider socio-political factors that may influence the development and/or delivery of the intervention (15).		
2. Report the purpose of the intervention development process.	Clearly describing the purpose of the intervention specifies what it sets out to achieve. The purpose may be informed by research priorities, for example those identified in systematic reviews, evidence gaps set out in practice guidance such as The National Institute for Health and Care Excellence or specific prioritisation exercises such as those undertaken with patients and practitioners through the James Lind Alliance.		
3. Report the target population for the intervention development process.	The target population is the population that will potentially benefit from the intervention – this may include patients, clinicians, and/or members of the public. If the target population is clearly described then readers will be able to understand the relevance of the intervention to their own research or practice. Health inequalities, gender and ethnicity are features of the target population that may be relevant to intervention development processes.		
4. Report how any published intervention development approach contributed to the development process	Many formal intervention development approaches exist and are used to guide the intervention development process (e.g. 6Squid (16) or The Person Based Approach to Intervention Development (17)). Where a formal intervention development approach is used, it is helpful to describe the process that was followed, including any deviations. More general approaches to intervention development also exist and have been categorised as follows (3):- Target Population-centred intervention development; evidence and theory-based intervention development; partnership intervention development; implementation-based intervention development; efficacy-based intervention development; step or phased-based intervention development; and intervention-specific intervention development (3). These approaches do not always have specific guidance that describe their use. Nevertheless, it is helpful to give a rich description of how any published approach was operationalised		
5. Report how evidence from different sources informed the intervention development process.	Intervention development is often based on published evidence and/or primary data that has been collected to inform the intervention development process. It is useful to describe and reference all forms of evidence and data that have informed the development of the intervention because evidence bases can change rapidly, and to explain the manner in which the evidence and/or data was used. Understanding what evidence was and was not available at the time of intervention development can help readers to assess transferability to their current situation.		
6. Report how/if published theory informed the intervention development process.	Reporting whether and how theory informed the intervention development process aids the reader's understanding of the theoretical rationale that underpins the intervention. Though not mentioned in the e-Delphi or consensus meeting, it became increasingly apparent through the development of our guidance that this theory item could relate to either existing published theory or programme theory		
7. Report any use of components from an existing intervention in the current intervention development process.	Some interventions are developed with components that have been adopted from existing interventions. Clearly identifying components that have been adopted or adapted and acknowledging their original source helps the reader to understand and distinguish between the novel and adopted components of the new intervention.		
8. Report any guiding principles, people or factors that were prioritised when making decisions during the intervention development process.	Reporting any guiding principles that governed the development of the application helps the reader to understand the authors' reasoning behind the decisions that were made. These could include the examples of particular populations who views are being considered when designing the intervention, the modality that is viewed as being most appropriate, design features considered important for the target population, or the potential for the intervention to be scaled up.		

For peer review only - http://bmjopen.bmj.com/site/about/guidelines.xhtml

Item description	Explanation	Page in manuscript where item is located	Other*
9. Report how stakeholders contributed to the intervention development process.	Potential stakeholders can include patient and community representatives, local and national policy makers, health care providers and those paying for or commissioning health care. Each of these groups may influence the intervention development process in different ways. Specifying how differing groups of stakeholders contributed to the intervention development process helps the reader to understand how stakeholders were involved and the degree of influence they had on the overall process. Further detail on how to integrate stakeholder contributions within intervention reporting are available (19).		
10. Report how the intervention changed in content and format from the start of the intervention development process.	Intervention development is frequently an iterative process. The conclusion of the initial phase of intervention development does not necessarily mean that all uncertainties have been addressed. It is helpful to list remaining uncertainties such as the intervention intensity, mode of delivery, materials, procedures, or type of location that the intervention is most suitable for. This can guide other researchers to potential future areas of research and practitioners about uncertainties relevant to their healthcare context.		
11. Report any changes to interventions required or likely to be required for subgroups.	Specifying any changes that the intervention development team perceive are required for the intervention to be delivered or tailored to specific sub groups enables readers to understand the applicability of the intervention to their target population or context. These changes could include changes to personnel delivering the intervention, to the content of the intervention, or to the mode of delivery of the intervention.		
12. Report important uncertainties at the end of the intervention development process.	Intervention development is frequently an iterative process. The conclusion of the initial phase of intervention development does not necessarily mean that all uncertainties have been addressed. It is helpful to list remaining uncertainties such as the intervention intensity, mode of delivery, materials, procedures, or type of location that the intervention is most suitable for. This can guide other researchers to potential future areas of research and practitioners about uncertainties relevant to their healthcare context.		
13. Follow TIDieR guidance when describing the developed intervention.	Interventions have been poorly reported for a number of years. In response to this, internationally recognized guidance has been published to support the high quality reporting of health care? interventions[5]and public health interventions[14]. This guidance should therefore be followed when describing a developed intervention.		
14. Report the intervention development process in an open access format.	Unless reports of intervention development are available people considering using an intervention cannot understand the process that was undertaken and make a judgement about its appropriateness to their context. It also limits cumulative learning about intervention development methodology and observed consequences at later evaluation, translation and implementation stages. Reporting intervention development in an open access (Gold or Green) publishing format increases the accessibility and visibility of intervention development research and makes it more likely to be read and used. Potential platforms for open access publication of intervention development include open access journal publications, freely accessible funder reports or a study web-page that details the intervention development process.		

*e.g. if item is reported elsewhere, then the location of this information can be stated here.

For peer review only
